# Continuous Transmission of Delayed Bit-Interleaved Coded Modulation with Capacity-Preserving Boundary Pilot Design †

**DOI:** 10.3390/e28101087

**Published:** 2026-10-05

**Authors:** Gou Hosoya

**Affiliations:** Department of Systems Engineering, Faculty of Systems Engineering, Osaka Sangyo University, Osaka 574-8530, Japan; hosoya@ge.osaka-sandai.ac.jp

**Keywords:** BICM, DBICM, CM capacity, PAM, QAM, LDPC code

## Abstract

Bit-interleaved coded modulation (BICM) with Gray labeling is widely used, yet its mismatched decoding metric keeps achievable rates below the coded modulation (CM) capacity, which multilevel coding attains only with multiple component codes. Delayed BICM (DBICM) bridges this gap with a single encoder and decoder by delaying coded bit subsequences so that already-decoded bits act as side information; for staircase delay vectors, its middle-region rate provably equals the CM capacity for any labeling. Under continuous transmission, however, initial and final codewords share symbols with pilot bits, and these boundary rates depend strongly on the pilot values. For pulse amplitude modulation, we design delay vectors and boundary pilots that keep every boundary rate at or above the middle-region capacity. For set-partitioning (SP) labeling, a closed-form minimum-shift pilot rule provably guarantees this condition for every staircase vector and SNR, whereas Gray labeling admits capacity-preserving pilots found by numerical search up to 32-PAM. Density evolution predicts gains approaching 1 dB over rate-matched BICM baselines for both labelings. Long continuous simulations, however, reveal severe inter-codeword error propagation under SP labeling, establishing Gray labeling as the practical choice. With per-level irregular LDPC codes, DBICM retains consistent threshold and waterfall gains at identical effective rates.

## 1. Introduction

Bit-interleaved coded modulation (BICM) [1] with Gray labeling [2,3] is the standard bandwidth-efficient transmission scheme in modern wireless systems [4], and its capacity is close to the coded modulation (CM) capacity [5,6,7]. The integration of BICM with advanced channel codes has accordingly received considerable attention [8,9,10,11,12]. BICM nevertheless employs a mismatched decoding metric [6], and its achievable rate remains below the CM capacity. Multilevel coding with multistage decoding (MLC/MSD) [13], typically combined with set-partitioning (SP) labeling, attains the CM capacity [14] and remains an active research topic [15]. It requires, however, *m* component codes of distinct rates for a $2^{m}$-ary constellation, whereas BICM operates with a single encoder and a single decoder.

Delayed BICM (DBICM) closes this gap while retaining the single-codec architecture [16,17,18,19]. Several codewords are transmitted concurrently, with a different delay applied to each label-bit subsequence, so that every symbol carries bits belonging to distinct codewords. A codeword is decoded once all of its subsequences have been received. The decisions on previously decoded codewords then serve as side information for the remaining levels of the symbols that they share, which raises the achievable rate above the BICM capacity. For an arbitrary labeling, the DBICM rate attains the CM capacity when the maximum delay satisfies $d_{\max}=m-1$ [19]. Smaller delays suffice for square QAM with product Gray labeling, for which $d_{\max}=m/2-1$ [19], and for APSK with Gray or pseudo-Gray labeling, for which $d_{\max}=m-2$ [20,21]. Convolutional polar coded modulation [22] exploits a similar temporal coupling but requires dedicated polar constructions, whereas DBICM is applicable to any binary linear block code.

The problem addressed in this paper is the following. The prior studies [19,20,21] evaluate the rate of a codeword located in the stationary interior of an infinitely long stream. An actual transmission, however, consists of a finite train of *T* codewords, and the delays leave bit positions unfilled in the first and the last $d_{\max}$ symbol blocks. These positions must be occupied by pilot bits, which restrict the active constellation and necessitate a re-normalization of the transmit power. The rates of the boundary codewords consequently depend on the pilot values, and may fall far below the middle-region capacity. DBICM relies on error-free side information in the same manner as MSD. A rate deficit at the boundary therefore causes decoding failures that propagate through the frame. The ISIT 2022 paper [21] defined boundary rates under all-zero pilots without power re-normalization, a setting in which the boundary symbols were free to draw on the outer, high-energy points of the constellation. The ISITA 2024 paper [23] introduced the unit-power model adopted here. The boundary rates under general pilots and the design of the pilots themselves nevertheless remained open.

This paper treats DBICM as a continuous transmission of *T* codewords. The rate of every codeword in the start, middle, and end regions is characterized, and the delay vector and the boundary pilots are designed such that no boundary codeword falls below the middle-region CM capacity. The analysis is conducted for one-dimensional PAM, to which square QAM with product labeling reduces dimension by dimension, and compares Gray labeling, which is the customary choice for BICM, with SP labeling, which is the customary choice for MLC/MSD. The contributions are fourfold.

1. For SP labeling, it is proved that a closed-form *minimum-shift* pilot rule keeps every boundary bit rate at or above its middle-region counterpart for every staircase delay vector and every SNR. For Gray labeling, no such structural guarantee exists, and capacity-preserving pilots are obtained by exhaustive search for constellations up to 32-PAM.
2. A position-dependent density evolution (DE) analysis shows that, under the designed pilots, Gray and SP labeling exhibit nearly identical thresholds, and that the gain over rate-matched BICM grows to approximately 1 dB for 32-PAM at a rate of 1/4.
3. Long finite-length simulations reveal an error-propagation effect that is invisible to both the capacity and the DE analyses. The performance of SP labeling degrades continuously with *T*, whereas that of Gray labeling is invariant to *T* and agrees with the DE prediction.
4. With irregular LDPC codes, the gain persists and amounts to 0.09 dB for 4-PAM and 0.55 dB for 8-PAM. The code is adapted to the delay by reusing the BICM degree profiles, which remain unchanged for 4-PAM and require only a permutation of the level assignment for 8-PAM.

The remainder of the paper is organized as follows. Section 2 and Section 3 introduce the system model and the operation of DBICM. Section 4 defines the pilots and the boundary regions, Section 5 derives the achievable rates, and Section 6 presents the pilot design. Section 7 and Section 8 report the DE analysis and the simulation results, and Section 9 concludes the paper.

## 2. System Model and Notation

Boldface letters denote vectors, and uppercase and lowercase letters denote random variables (RVs) and their realizations, respectively. The number of bits per symbol is *m* with $M≜2^{m}$, the codeword length is $n_{c}$, which is a multiple of *m*, and the number of symbols per codeword is $N_{s}≜n_{c}/m$.

### 2.1. Constellation and Channel

An *M*-PAM constellation X⊂R with an arbitrary bijective labeling $\mu :{\left\{0,1\right\}}^{m}\to X$, x=μ(b), is considered. The transmitted point *X* is equiprobable, that is,

(1) Pr{X=x}=1/M,x∈X,

or, equivalently, the label bits are independent and uniformly distributed, and the power is normalized as

(2) $$E[X^{2}]=1.$$

Probabilistic and geometric shaping [24,25] act on the input distribution or on the point geometry in order to close the gap between the CM capacity and the Gaussian capacity. DBICM, by contrast, closes the gap between the BICM rate and the CM capacity, which is left untouched by shaping. The two mechanisms are therefore orthogonal and may be combined. The uniform input is retained in this paper so that the delay vector and the pilot values remain the only design variables and their effect is isolated. The channel is Y=X+Z with $Z∼N(0,\sigma^{2})$, so that $N_{0}=2\sigma^{2}$ and $E_{s}/N_{0}=1/\left(2\sigma^{2}\right)$ with $E_{s}=1$. All SNR values, thresholds, and axes are expressed in terms of $E_{s}/N_{0}$, whereas the ratio $1/\sigma^{2}=2E_{s}/N_{0}$ appears only in the Gaussian capacity of Remark 4. The results carry over to square $M^{2}$-QAM with product labeling, which decomposes into two independent *M*-PAM constellations of energy 1/2 each. Every rate, threshold, and margin reported at a given $E_{s}/N_{0}$ therefore applies to one real dimension of a QAM constellation whose total symbol SNR is higher by $10\log_{10}2\approx 3.01$ dB.

### 2.2. Bit Labels

The label of a symbol is

(3) $$B=(B_{0},B_{1},\ldots ,B_{m-1}),$$

where $B_{0}$ is referred to as the MSB and $B_{m-1}$ as the LSB. These names designate coordinate positions and do not imply a significance intrinsic to an arbitrary μ. The bit channels $B_{i}\to Y$ generally carry different mutual information, and it is this asymmetry that DBICM exploits.

**Figure 1:**
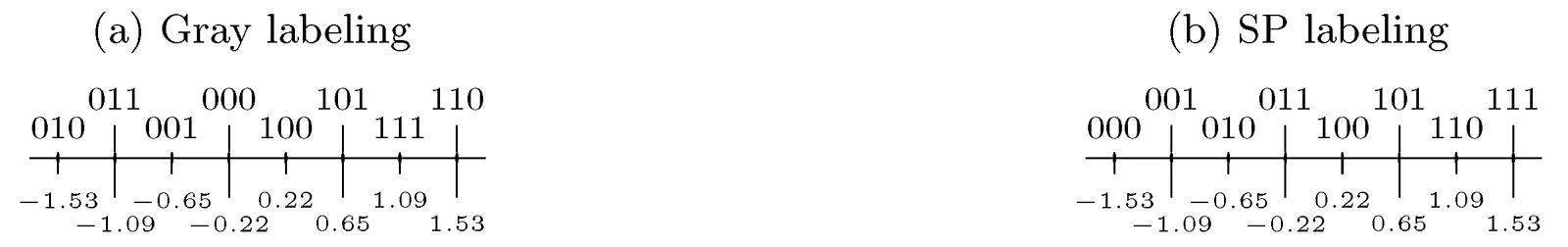
The two 8-PAM labelings used in this paper: (**a**) Gray and (**b**) SP (natural binary). Bit strings are $\left(b_{0}b_{1}b_{2}\right)$, and the axis shows normalized amplitudes.

Figure 1 shows the two labelings compared in this paper for 8-PAM. For reproducibility at an arbitrary $M=2^{m}$, the points are indexed as $x_{n}=\left(2n-M+1\right)\kappa$ for n∈{0,…,M−1}, where $\kappa =\sqrt{3/(M^{2}-1)}$. Gray labeling [3], shown in Figure 1a, is the reflected construction. The bit $B_{0}$ equals 1 if and only if $x_{n}>0$, and, writing $|x_{n}|=\left(2\ell +1\right)\kappa$, the bits $\left(B_{1},\ldots ,B_{m-1}\right)$ form the (m−1)-bit binary expansion of ℓ⊕⌊ℓ/2⌋, with $B_{1}$ as its most significant bit. SP labeling [13,26], shown in Figure 1b, is the natural binary map $n=\sum_{i=0}^{m-1}B_{i}2^{m-1-i}$, in which every prefix $\left(b_{0},\ldots ,b_{i-1}\right)$ selects a Euclidean translation of a smaller PAM constellation. This property underpins the analysis of Section 6.2.

### 2.3. Coded Modulation Capacity

The reference for all rates in this paper is the CM capacity

(4) $$C_{\mathrm{CM}}≜I\left(X;Y\right)=\frac{1}{M}\sum_{x\in X}\int p_{Y|X}\left(y|x\right)\log_{2}\frac{p_{Y|X}\left(y|x\right)}{p_{Y}\left(y\right)}\;dy,$$

which, under (1), is determined by X and the SNR alone. The CM capacity is therefore invariant to the labeling μ. Every bit-level quantity introduced below, namely the BICM rate and each per-level term of the DBICM rates, depends on μ. The delay design thus approaches a labeling-independent target through labeling-dependent terms.

## 3. Delayed BICM (DBICM)

**Figure 2:**
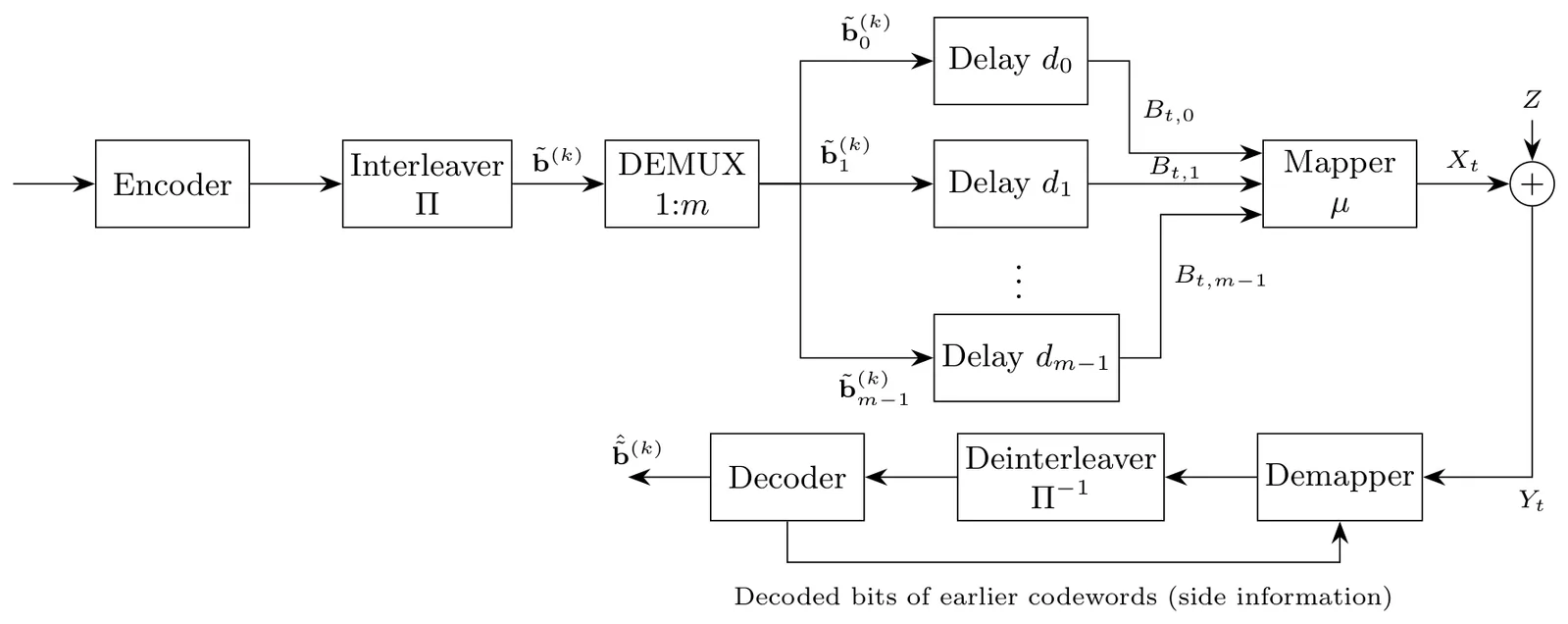
Block diagram of the coded DBICM system. The interleaved codeword ${\tilde{b}}^{\left(k\right)}$ is demultiplexed into *m* subsequences, the *i*-th of which is delayed by $d_{i}$ codeword blocks before mapping. Decoded bits of earlier codewords are fed back to the demapper.

**Figure 3:**
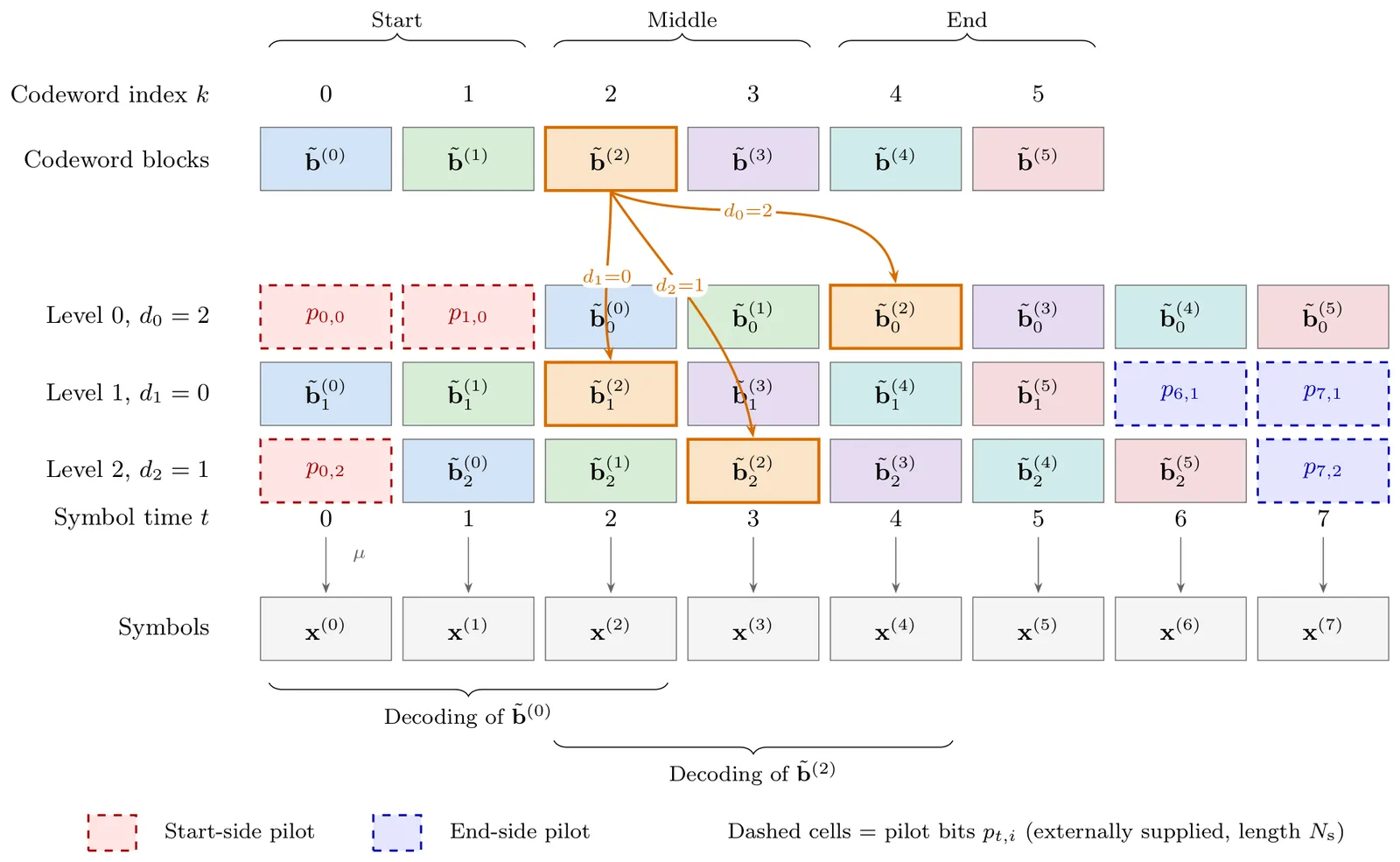
DBICM system model with m=3, d=(2,0,1), and T=6. The cell colors serve only to distinguish the codewords.

Figure 2 shows the block diagram of the system, and Figure 3 shows its timing structure for the running example with m=3, d=(2,0,1), and T=6.

### 3.1. Codewords, Delay Vector, and Delay Operation

Let ${\tilde{b}}^{\left(k\right)}\in {\left\{0,1\right\}}^{n_{c}}$, k∈{0,…,T−1} denote the *k*-th encoded and interleaved codeword of rate *R*. It is demultiplexed into *m* subsequences ${\tilde{b}}_{i}^{\left(k\right)}=\left({\tilde{b}}_{0,i}^{\left(k\right)},\ldots ,{\tilde{b}}_{N_{s}-1,i}^{\left(k\right)}\right)$, one for each label position *i*. The interleaver is immaterial to the rate analysis and is left unspecified. Since the channel is memoryless, the within-block index *j* is suppressed, and ${\tilde{B}}_{i}^{\left(k\right)}$ denotes a generic bit of ${\tilde{b}}_{i}^{\left(k\right)}$.

The delay vector is $d=(d_{0},\ldots ,d_{m-1})$ with $d_{i}\in Z_{\geq 0}$ and $d_{\max}≜\max_{i}d_{i}$, on which the admissibility condition

(5) $$\left\{d_{0},\ldots ,d_{m-1}\right\}=\left\{0,1,\ldots ,d_{\max}\right\}$$

is imposed. This condition entails no loss of generality, because a common positive offset can be subtracted and a skipped value can be collapsed without altering the decoding order or the side-information structure, while $d_{\max}$ is reduced. The transmission of *T* codewords then occupies $T_{s}=T+d_{\max}$ symbol blocks, and the effective rate $R_{\mathrm{eff}}$ is defined in (17).

Let $X_{t}$ and $B_{t,i}$ denote the RVs of a representative symbol and of its level-*i* bit at block time $t\in \{0,\ldots ,T_{s}-1\}$, where $X_{t}=\mu \left(B_{t,0},\ldots ,B_{t,m-1}\right)$. The delay operation places the level-*i* subsequence of codeword $t-d_{i}$ at time *t*, that is,

(6) $$B_{t,i}={\tilde{B}}_{i}^{\left(t-d_{i}\right)},\;t-d_{i}\in \left\{0,\ldots ,T-1\right\}.$$

If $t-d_{i}\notin \left\{0,\ldots ,T-1\right\}$, no codeword is available for the position in question. This situation arises in every finite transmission, namely in the first and the last $d_{\max}$ blocks, and the position is occupied by a *pilot bit*. The formalization and the design of the pilot bits are the subject of Section 4 and Section 6. The block at time *t* produces the symbol sequence

(7) $$x^{\left(t\right)}=\left(x_{0}^{\left(t\right)},\ldots ,x_{N_{s}-1}^{\left(t\right)}\right),\;x_{j}^{\left(t\right)}\in X,$$

which is shown at the bottom of Figure 3 and of which $X_{t}$ is the generic per-symbol RV.

### 3.2. Decoding Schedule and Delayed Feedback

Codeword *k* becomes decodable at time $k+d_{\max}$, and the codewords are consequently decoded in the order k=0,1,…,T−1, as indicated by the braces in Figure 3. Once codeword *k* has been decoded, the a posteriori log-likelihood ratios (LLRs) of its bits are fed back to the demapper, as indicated by the arrow in Figure 2, and serve as prior information for the symbols that it shares with later codewords. For the symbol carrying the level-*i* bit of codeword *k*, the co-located levels *j* with $d_{j}>d_{i}$ belong to earlier codewords and are available in this manner. The rate analysis of Section 5 conditions on these bits under the assumption of error-free decisions, whereas the effect of erroneous feedback is evaluated by simulation in Section 8.

**Remark** **1** (Relation to BICM-ID)**.** *BICM-ID exchanges soft extrinsic information iteratively between the demapper and the decoder within a single codeword, whereas DBICM passes decisions forward across codewords without iterating on the same codeword. The two mechanisms are orthogonal and may be combined [18], which lies outside the scope of this paper.*

**Remark** **2** (Decoding complexity relative to BICM)**.** *Every codeword is decoded exactly once, as in BICM, so that the decoding cost is unchanged and the additional effort is confined to the demapper. Under BICM, the level-i LLR sums the likelihoods $p_{Y|X}\left(y|x\right)$ over the $2^{m-1}$ labels that agree with the target bit. Under DBICM, the co-located levels j with $d_{j}>d_{i}$ carry decoded or pilot bits whose a posteriori LLRs are converted into probabilities $q_{j}\left(\cdot \right)$ and enter the sum as a non-uniform prior, so that the summands become $p_{Y|X}\left(y|x\right)\prod_{j:d_{j}>d_{i}}q_{j}\left(b_{j}\right)$. The number of metric evaluations per symbol equals that of BICM but is spread over m decoding instants. The level with delay $d_{i}$ receives $m-1-d_{i}$ priors, and m2 prior insertions per symbol are required in total, so that the demapping cost grows by a factor of at most (m+1)/2, independently of T and of the code length, provided that the $2^{m}$ likelihoods of each received symbol are computed once and cached. The receiver buffers $d_{\max}$ symbol blocks and decodes each codeword $d_{\max}$ blocks after the arrival of its first subsequence, where $d_{\max}\leq m-1$ is independent of T.*

### 3.3. Special Delay Settings

For d=0, DBICM reduces to BICM, and its rate equals the BICM capacity. For a *staircase* delay vector, that is, a permutation of (0,1,…,m−1) with $d_{\max}=m-1$, DBICM is equivalent to MLC/MSD [13,14]. The level-*i* term of codeword *k* is conditioned on the previously decoded levels $\left\{j:d_{j}>d_{i}\right\}$. Since the delays are distinct, these sets are nested, so that the chain rule applies for every bijective μ and the middle-region rate attains the CM capacity (Proposition 1). For the reversed staircase

(8) d=(m−1,m−2,…,1,0),

the levels are decoded in the order 0,1,…,m−1, so that the level-*i* term is conditioned on levels 0,…,i−1 and the sum of the *m* terms is the ordinary chain-rule expansion of I(X;Y), which is stated formally in Proposition 1. The conditioning bits belong physically to earlier codewords but are statistically identical to those of codeword *k* by the stationarity of the middle region. Delay vectors between these two extremes interpolate between the BICM capacity and the CM capacity.

## 4. Pilot Bits and Boundary Regions

Under continuous transmission, the symbols near the two ends of the frame contain bit positions for which no codeword is available. These positions are occupied by pilot bits, which correspond to the dashed cells in Figure 3. This section collects in Table 1 the notation required to describe the location of the pilots, the codewords that they affect, and the manner in which they restrict and re-normalize the transmitted constellation. The running example of Figure 3, with m=3, d=(2,0,1), and T=6, is used throughout.

### 4.1. Index Correspondence, Pilot Index Sets, and Regions

The achievable rate is evaluated for each codeword k∈{0,…,T−1}, whereas the symbols are transmitted along the extended time axis $t\in \{0,\ldots ,T_{s}-1\}$, with $T_{s}=T+d_{\max}$. Since level *i* is delayed by $d_{i}$, the level-*i* subsequence of codeword *k* is transmitted at the time

(9) $$t=\psi_{i}\left(k\right)≜k+d_{i},\;i\in \left\{0,1,\ldots ,m-1\right\}.$$

Pilots are associated with symbol times rather than with codewords. At time *t*, the pilot-fixed bit positions are the levels whose codeword index $t-d_{j}$ falls outside the transmitted range, that is,

(10) $$I_{t}≜\left\{\;j\in \left\{0,\ldots ,m-1\right\}\;:\;t-d_{j}\notin \left\{0,\ldots ,T-1\right\}\;\right\},$$

and the corresponding pilot values $p_{t,j}\in \left\{0,1\right\}$, $j\in I_{t}$, are collected in the vector $p_{t}\in {\left\{0,1\right\}}^{|I_{t}|}$. By (5), the set $I_{t}$ is nonempty only at the start times $t<d_{\max}$ and at the end times t≥T, and it is empty at all intermediate times. The codewords are partitioned accordingly into the start, middle, and end regions $K_{\mathrm{start}}$, $K_{\mathrm{mid}}$, and $K_{\mathrm{end}}$ listed in Table 1, of which only the codewords in $K_{\mathrm{start}}\cup K_{\mathrm{end}}$ share a symbol time with a pilot. In a practical block, each scalar $p_{t,j}$ fixes the entire level-*j* subsequence of length $N_{s}$ at time *t* to the all-zero or to the all-one sequence.

### 4.2. Sub-Constellations and Re-Normalization

**Figure 4:**
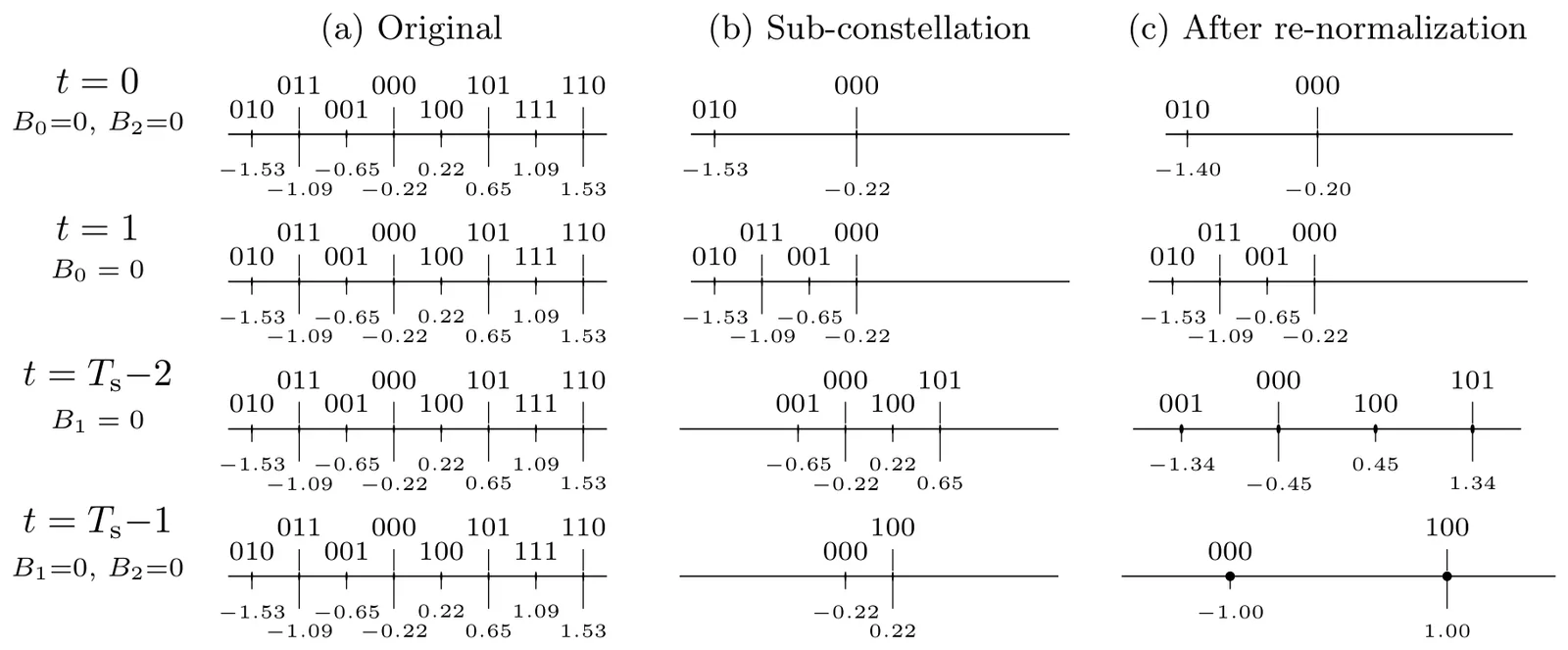
Sub-constellations and re-normalization for Gray-labeled 8-PAM with d=(2,0,1) and all-zero pilots. Rows are the boundary times $t\in \{0,1,T_{s}-2,T_{s}-1\}$, with the pilot-fixed bits indicated below each time. Columns show (**a**) the full constellation X, (**b**) the retained sub-constellation $X_{t}\left(p_{t}\right)$, and (**c**) its re-normalization to unit average power.

Fixing the positions in $I_{t}$ restricts the symbol at time *t* to the sub-constellation $X_{t}\left(p_{t}\right)$ of Table 1, whose average energy $E_{t}\left(p_{t}\right)$ generally differs from one. In order to maintain a unit average transmit power at every symbol time, the transmitted symbol is $X_{t}=X^{\prime }/\gamma_{P}$, where $X^{\prime }$ is uniformly distributed on $X_{t}\left(p_{t}\right)$ and $\gamma_{P}=\sqrt{E_{t}\left(p_{t}\right)}$ (Table 1). Both the active alphabet and its scaling therefore depend on the pilot values. Figure 4 illustrates this dependence for Gray-labeled 8-PAM with d=(2,0,1) and all-zero pilots. The four boundary times have $I_{0}=\left\{0,2\right\}$, $I_{1}=\left\{0\right\}$, $I_{T_{s}-2}=\left\{1\right\}$, and $I_{T_{s}-1}=\left\{1,2\right\}$. The surviving points are compressed when their energy exceeds one, as in the case where $E_{0}=25/21$ at t=0, and are expanded when their energy falls below one, as in the case where $E_{T_{s}-1}=1/21$, which maps the innermost pair onto {−1,+1}.

## 5. Achievable Rates

### 5.1. DBICM Achievable Rate in the Middle Region

Recall from Section 4.1 that the *i*-th label bit of codeword *k* is transmitted at symbol time $\psi_{i}\left(k\right)≜k+d_{i}$. A codeword *k* belongs to the middle region, $k\in K_{\mathrm{mid}}$, when none of the label bits sharing a symbol time with any bit of codeword *k* is a pilot bit. The start and end regions $K_{\mathrm{start}}$ and $K_{\mathrm{end}}$ collect the remaining boundary codewords.

For $k\in K_{\mathrm{mid}}$, the achievable rate assigned to codeword *k* can be evaluated independently of the pilot bits. Each level-*i* bit is received at symbol time $\psi_{i}\left(k\right)$ alongside the side information consisting of the label bits in the same symbol that belong to already-decoded codewords. A bit at level *j* of the symbol at time *t* belongs to codeword $t-d_{j}$, which is decoded prior to codeword $t-d_{i}$ if and only if $d_{j}>d_{i}$. The corresponding side-information set is therefore defined as

(11) $$H_{t,i}\left(d\right)≜\left\{\;B_{t,j}\;:\;d_{j}>d_{i}\;\right\},$$

which makes the dependence on the delay vector explicit. Where no confusion arises, we suppress the argument and write simply $H_{t,i}$. With this notation, the achievable rate of codeword $k\in K_{\mathrm{mid}}$ is given by

(12) $$C_{\mathrm{DBICM}}^{\mathrm{mid}}\left(k;d\right)≜\sum_{i=0}^{m-1}I\;\left(B_{\psi_{i}\left(k\right),i};Y_{\psi_{i}\left(k\right)}\;|\;H_{\psi_{i}\left(k\right),i}\left(d\right)\right).$$

For $k\in K_{\mathrm{mid}}$, every symbol time $\psi_{i}\left(k\right)=k+d_{i}$ falls within $\left\{d_{\max},\ldots ,T-1\right\}$. These are precisely the pilot-free times at which $I_{t}=\emptyset$ and the channel input is drawn from the original constellation X. By the stationarity of the channel and the input process over these time slots, each summand depends solely on the bit level *i* and not on the codeword index *k*. Consequently, $C_{\mathrm{DBICM}}^{\mathrm{mid}}\left(k;d\right)$ is identical for every $k\in K_{\mathrm{mid}}$, and it can be evaluated at any representative pilot-free symbol time:

(13) $$C_{\mathrm{DBICM}}^{\mathrm{mid}}\left(d\right)≜C_{\mathrm{DBICM}}^{\mathrm{mid}}\left(k;d\right)=\sum_{i=0}^{m-1}I\;\left(B_{t,i};Y_{t}\;|\;H_{t,i}\left(d\right)\right),\;t\in \left\{d_{\max},\ldots ,T-1\right\}.$$

It is convenient to define the individual per-level summands as

(14) $$C_{i}^{\mathrm{mid}}\left(d\right)≜I\;\left(B_{t,i};Y_{t}\;|\;H_{t,i}\left(d\right)\right),\;t\in \left\{d_{\max},\ldots ,T-1\right\},$$

which, by the same stationarity argument, does not depend on the choice of the representative pilot-free time *t*, so that $C_{\mathrm{DBICM}}^{\mathrm{mid}}\left(d\right)=\sum_{i=0}^{m-1}C_{i}^{\mathrm{mid}}\left(d\right)$. With (14), the middle-region rate reference carries no free temporal index. From here on, the index *t* always denotes a transmission (symbol) time; whenever it is associated with a pair (k,i), it implicitly refers to $t=\psi_{i}\left(k\right)$ at which that specific bit is transmitted.

**Proposition** **1** (Staircase delay vectors attain the CM capacity)**.** *Let d be any permutation of (0,1,…,m−1). Then, for every SNR and every labeling μ,*

(15) $$C_{\mathrm{DBICM}}^{\mathrm{mid}}\left(d\right)=\sum_{i=0}^{m-1}I\;\left(B_{t,i};Y_{t}\;|\;H_{t,i}\left(d\right)\right)=I\left(X;Y\right).$$

**Proof.**  Since all delay components are distinct, the conditioning sets in (11) are strictly nested and, together with the target bit $B_{t,i}$, exhaust all *m* label bits. The assertion then follows directly from the chain rule of mutual information, which holds because μ is a bijection. □

The reversed staircase d=(m−1,…,1,0) of (8), for which $H_{t,i}\left(d\right)=\left\{B_{t,0},\ldots ,B_{t,i-1}\right\}$, corresponds to the chain-rule expansion in the natural order of the levels.

### 5.2. CM Capacity-Achieving Delay Vectors

**Definition** **1** (CM capacity-achieving delay–pilot pair)**.** *A delay vector d together with a pilot assignment $\left\{p_{t,i}\right\}$ is* CM capacity-achieving *(CMCA) for a labeled constellation (X,μ) if, at every SNR:*

*(i)*  *The middle-region rate equals the CM capacity,*
  (16) $$C_{\mathrm{DBICM}}^{\mathrm{mid}}\left(d\right)=I\left(X;Y\right).$$
*(ii)*  *No boundary codeword is a bottleneck, that is, the achievable rate of every boundary codeword $k\in K_{\mathrm{start}}\cup K_{\mathrm{end}}$, formalized in Section 5.3, is no smaller than $C_{\mathrm{DBICM}}^{\mathrm{mid}}\left(d\right)$.*

*The pair is termed* bit-level *CMCA if, in addition, every per-level summand of every boundary codeword is no smaller than the corresponding middle-region summand. A delay vector itself is termed CMCA, or bit-level CMCA, when it admits a pilot assignment with the respective property.*

Condition (i) is settled by Proposition 1, since every staircase permutation of (0,1,…,m−1) satisfies it for an arbitrary labeling μ. Condition (ii) is the substantial requirement, because the boundary terms depend on the pilot values through the restriction and re-normalization of the constellation. Condition (ii) is also the operationally relevant one, since the density evolution threshold of Section 7 is governed by the weakest codeword. The bit-level property is a stronger sufficient condition, which is convenient to establish analytically. For SP labeling, Section 6.2 proves that every staircase delay vector is bit-level CMCA under a closed-form pilot rule. For Gray labeling, no such structural guarantee exists. Section 6.3 shows numerically that the bit-level property is unattainable beyond 8-PAM, whereas the codeword-level property is attained for every constellation size considered.

Regarding condition (i), a natural question arises as to whether the middle-region equality can also hold for delay vectors with a smaller maximum delay, since $d_{\max}$ dictates both the decoding latency and the boundary overhead. Transmitting *T* codewords occupies $T+d_{\max}$ symbol times, so that the effective code rate of the scheme is given by

(17) $$R_{\mathrm{eff}}\;=\;R\;\frac{T}{T+d_{\max}}.$$

Three cases are either known or immediate regarding the middle-region condition (i):

(a) For 4-PAM (m=2), the question is trivial because the only delay vectors with distinct entries are the two staircases (0,1) and (1,0). The full chain rule is therefore always attained at the minimum possible delay $d_{\max}=m-1=1$, and 4-PAM serves as a check for the numerical pipeline.
(b) For square $2^{m}$-QAM with product Gray labeling and even *m*, the middle-region equality already holds at $d_{\max}=m/2-1$ [19]. In this case only, *m* denotes the total number of bits per QAM symbol in accordance with [19], whereas elsewhere in this paper, *m* counts the bits per one-dimensional PAM symbol (Section 2.1); the constituent PAM per real dimension thus carries m/2 bits.
(c) For APSK constellations with Gray or pseudo-Gray labeling, $d_{\max}=m-2$ suffices [20,21].

Case (b) can also be viewed as a corollary of the one-dimensional analysis, since a square $2^{m}$-QAM constellation with a product labeling factors into two independent $2^{m/2}$-PAM constellations (one per real dimension), as noted in Section 2.1. Its CMCA condition therefore reduces to that of the constituent $2^{m/2}$-PAM applied per dimension, whose staircase permutation yields $d_{\max}=m/2-1$. For this reason, we treat one-dimensional *M*-PAM as the general object of study and do not evaluate QAM separately, with the corresponding QAM results following directly on a per-real-dimension basis. For one-dimensional *M*-PAM itself, by contrast, no delay vector with $d_{\max}<m-1$ satisfies condition (i). If two levels *i* and *j* share the same delay, neither is conditioned on the other, and the sum of the two bit-level terms falls short of the chain-rule value by $I(B_{i};B_{j}|Y,H)$, where H denotes the common side information. This quantity is strictly positive at every finite SNR for a bijective labeling of a real constellation, because the two bits, independent a priori, become dependent given the noisy observation. The staircase vectors are therefore the only CMCA candidates for PAM.

### 5.3. DBICM Achievable Rate in the Start/End Regions

At a boundary symbol time *t*, the pilots impose the constraint

(18) $$P_{t}\left(p_{t}\right)≜\left\{\;B_{t,j}=p_{t,j}\;:\;j\in I_{t}\;\right\},$$

and the pair (k,i), which is transmitted at $t=\psi_{i}\left(k\right)$, is subject to the constraint

(19) $$P_{k,i}≜P_{\psi_{i}\left(k\right)}\left(p_{\psi_{i}\left(k\right)}\right),$$

which is empty whenever no pilot is present at the time $\psi_{i}\left(k\right)$.

**Definition** **2** (Re-normalized sub-constellation)**.** *For a pilot constraint $P=P_{t}\left(p_{t}\right)$, let $\gamma_{P}≜\sqrt{E_{t}\left(p_{t}\right)}$ denote the root-mean-square amplitude of the raw sub-constellation $X_{t}\left(p_{t}\right)$ of Section 4.2. The re-normalized sub-constellation is defined as*

(20) $$X_{P}≜\left\{\;x/\gamma_{P}\;:\;x\in X_{t}\left(p_{t}\right)\;\right\},\;E\left[X^{2}\right]=1\;\;\mathrm{for}\;X∼\mathrm{Unif}\left(X_{P}\right),$$

*with $\gamma_{\emptyset }=1$ and $X_{\emptyset }=X$.*

The mutual information with the channel input uniformly distributed on $X_{P}$ is denoted by $I_{X_{P}}\left(\cdot \;;\cdot |\cdot \right)$. The subscript encodes pilot fixing, which is an alphabet restriction followed by re-normalization, whereas the conditioning bar encodes conditioning on random variables. The two operations are thereby kept notationally separate.

The pilots co-located with a target bit lie on opposite sides of the decoding order at the two boundaries, as stated in the following lemma.

**Lemma** **1** (Location of pilot positions relative to the target)**.** *Let (k,i) be a boundary pair and $t=\psi_{i}\left(k\right)$. Then, $I_{t}\subseteq \left\{\;j:\;d_{j}>d_{i}\;\right\}$ at a start time and $I_{t}\subseteq \left\{\;j:\;d_{j}<d_{i}\;\right\}$ at an end time, irrespective of k.*

**Proof.**  If $t-d_{j}<0$, then $d_{j}>t=k+d_{i}\geq d_{i}$, which occurs only for $t<d_{\max}$. If $t-d_{j}>T-1$, then $d_{j}<t-\left(T-1\right)=d_{i}-\left(T-1-k\right)\leq d_{i}$, which occurs only for t≥T. □

Start-boundary pilots therefore occupy positions that would supply decoded side information in the middle region, whereas end-boundary pilots occupy positions that are marginalized in any case. The pilot positions must consequently be removed from the middle-region set $H_{t,i}\left(d\right)$ of (11) at the start boundary, which yields the boundary side-information set

(21) $$H_{t,i}^{\mathrm{bd}}\left(d\right)≜H_{t,i}\left(d\right)\setminus \left\{\;B_{t,j}:\;j\in I_{t}\;\right\}=\left\{\;B_{t,j}:\;d_{j}>d_{i},\;j\notin I_{t}\;\right\}.$$

At a start time, this set equals $\left\{B_{t,j}:d_{i}<d_{j}\leq t\right\}$, and at an end time, it coincides with $H_{t,i}\left(d\right)$ by Lemma 1.

**Definition** **3** (DBICM achievable rate in the boundary regions)**.** *For $k\in K_{\mathrm{start}}\cup K_{\mathrm{end}}$ and a given pilot assignment,*

(22) $$C_{\mathrm{DBICM}}\left(k;d,\left\{p_{t,i}\right\}\right)≜\sum_{i=0}^{m-1}I_{X_{P_{k,i}}}\;\left(B_{\psi_{i}\left(k\right),i};\;Y_{\psi_{i}\left(k\right)}\;|\;H_{\psi_{i}\left(k\right),i}^{\mathrm{bd}}\left(d\right)\right).$$

Depending on whether $P_{k,i}$ and $H_{\psi_{i}\left(k\right),i}^{\mathrm{bd}}$ are empty, each summand in (22) assumes one of three forms. It is a *fixing-only* term (F) when all co-located bits are pilots, and a *mixed* term (M) when pilots and decoded side information coexist. It is a *conditioning-only* term (C) when no pilot is present at the time in question, in which case it coincides with the middle-region summand of (14). Fixing acts on the alphabet through restriction and re-normalization, whereas conditioning acts only on the probability law and leaves the alphabet and its scaling unchanged. This distinction is maintained throughout the pilot design of Section 6.

**Figure 5:**
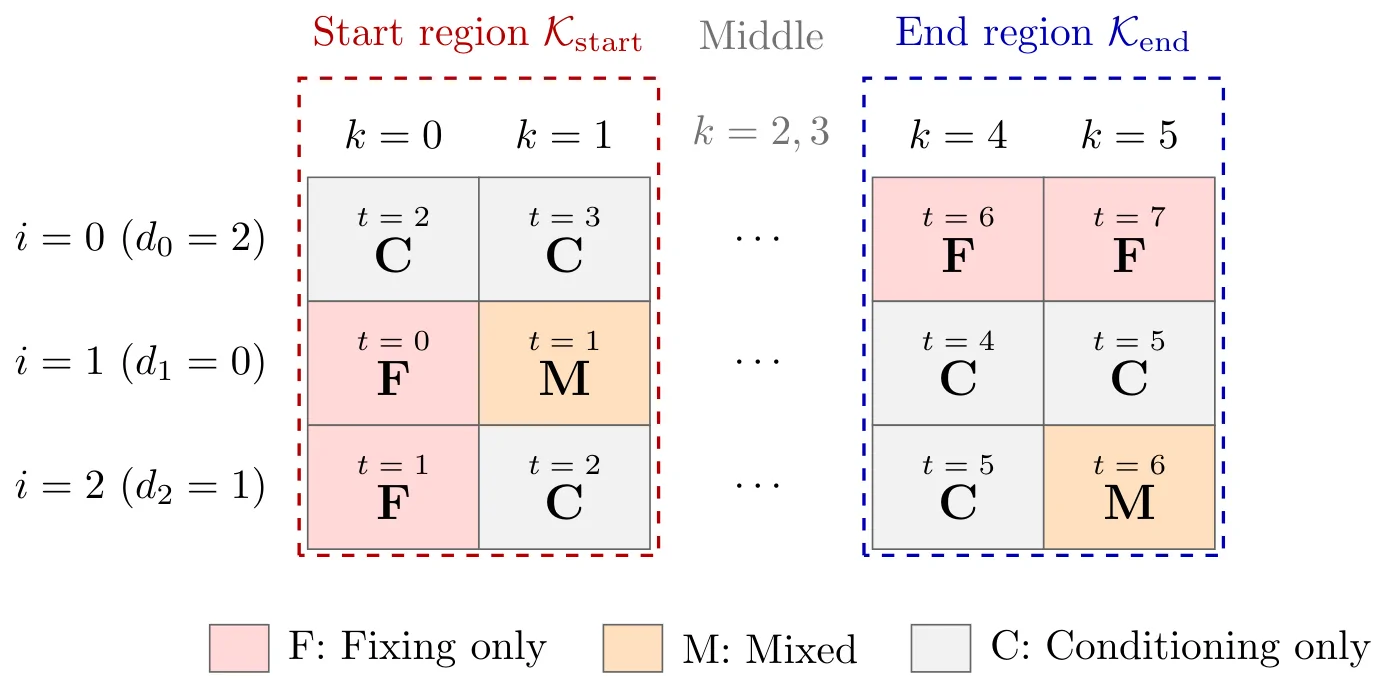
Classification of the boundary rate terms for m=3, d=(2,0,1), and T=6. Each cell is a pair (k,i) and shows the transmission time $t=\psi_{i}\left(k\right)$ and the form of its summand in (22): fixing only (F), mixed (M), or conditioning only (C). The middle-region codewords k∈{2,3} contribute only C terms.

**Example** **1** (Boundary terms of the running example)**.** *For m=3, d=(2,0,1), and T=6, Figure 5 classifies all boundary pairs. In each boundary region, exactly two pairs are fixing-only, one pair is mixed, and the remaining pairs are conditioning-only. For instance, the pair (k,i)=(1,1) is transmitted at t=1 with $I_{1}=\left\{0\right\}$ and $H_{1,1}^{\mathrm{bd}}=\left\{B_{1,2}\right\}$, which yields the mixed term $I_{X_{P_{1,1}}}\left(B_{1,1};Y_{1}|B_{1,2}\right)$. The pair (k,i)=(0,1), by contrast, is transmitted at t=0 with $I_{0}=\left\{0,2\right\}$, which exhausts $\left\{j:d_{j}>d_{1}\right\}$ and yields the fixing-only term $I_{X_{P_{0,1}}}\left(B_{0,1};Y_{0}\right)$.*

### 5.4. Dependence of Boundary Rates on Pilot Values

**Proposition** **2** (Dependence on the delay vector and pilot values)**.** *Fix an admissible delay vector d. For $k\in K_{\mathrm{mid}}$, the rate of codeword k equals $C_{\mathrm{DBICM}}^{\mathrm{mid}}\left(d\right)$ in (13) and is independent of k and of the pilots. For $k\in K_{\mathrm{start}}\cup K_{\mathrm{end}}$, the rate in (22) depends on the pilot values through the constraints $P_{k,i}$, and hence on $p_{\psi_{i}\left(k\right)}$ for every level i.*

**Proof.**  In the middle region, $I_{\psi_{i}\left(k\right)}=\emptyset$ for all *i*, so that every summand is conditioning-only and the stationarity argument of Section 5.1 applies. In the boundary regions, the pilot values enter only through the subscripts $X_{P_{k,i}}$, where $P_{k,i}$ is given by (18) and (19). □

Since the *m* label positions of a boundary time *t* belong to the *m* distinct codewords $t-d_{0},\ldots ,t-d_{m-1}$, a single pilot vector $p_{t}$ constrains several codewords, and a single boundary codeword collects constraints from *m* different times. The selection of the pilots is therefore a joint problem over the entire family $\left\{p_{t,j}\right\}$, and the design objective of Section 6.1 is formulated accordingly.

## 6. Pilot Design in the Boundary Regions

The boundary codewords need not become bottlenecks, because the pilot values are free design parameters. This section formulates the design objective, solves it in closed form for SP labeling, and solves it by exhaustive numerical search for Gray labeling.

### 6.1. Design Objective

Let

(23) $$D_{\mathrm{bd}}≜K_{\mathrm{bd}}\times \left\{0,\ldots ,m-1\right\},\;K_{\mathrm{bd}}≜K_{\mathrm{start}}\cup K_{\mathrm{end}},$$

denote the boundary pairs, and define the per-bit and per-codeword rate margins

(24) $$\begin{array}{cc} \Delta_{k,i}\left(d,\left\{p_{t,i}\right\}\right) & ≜I_{X_{P_{k,i}}}\;\left(B_{\psi_{i}\left(k\right),i};Y_{\psi_{i}\left(k\right)}\;|\;H_{\psi_{i}\left(k\right),i}^{\mathrm{bd}}\left(d\right)\right)-C_{i}^{\mathrm{mid}}\left(d\right), \end{array}$$

(25) $$\begin{array}{cc} \Delta_{k}\left(d,\left\{p_{t,i}\right\}\right) & ≜C_{\mathrm{DBICM}}\left(k;d,\left\{p_{t,i}\right\}\right)-C_{\mathrm{DBICM}}^{\mathrm{mid}}\left(d\right)=\sum_{i=0}^{m-1}\Delta_{k,i}\left(d,\left\{p_{t,i}\right\}\right). \end{array}$$

In terms of Definition 1, the pair $\left(d,\left\{p_{t,i}\right\}\right)$ is CMCA if $\Delta_{k}\geq 0$ for all $k\in K_{\mathrm{bd}}$ and all SNRs, and bit-level CMCA if $\Delta_{k,i}\geq 0$ for all $\left(k,i\right)\in D_{\mathrm{bd}}$. Only the pilot values at the boundary times, namely those with $I_{t}\neq \emptyset$, are optimization variables.

Only violations of the target are penalized. Let Δ(·;s) denote the margin at $E_{s}/N_{0}=s$, and let the margins be evaluated over a finite SNR grid S. The two penalties are defined as

(26) $$\begin{array}{cc} J_{\mathrm{bit}}\left(\{p_{t,i}\}\right) & ≜\sum_{s\in S}\sum_{\left(k,i\right)\in D_{\mathrm{bd}}}\min\left\{0,\;\Delta_{k,i}\left(d,\left\{p_{t,i}\right\};s\right)\right\}, \end{array}$$

(27) $$\begin{array}{cc} J_{\mathrm{cw}}\left(\{p_{t,i}\}\right) & ≜\sum_{s\in S}\sum_{k\in K_{\mathrm{bd}}}\min\left\{0,\;\Delta_{k}\left(d,\left\{p_{t,i}\right\};s\right)\right\}. \end{array}$$

Both penalties are nonpositive. On the grid, $J_{\mathrm{cw}}=0$ holds if and only if the pair is CMCA, and $J_{\mathrm{bit}}=0$ holds if and only if the pair is bit-level CMCA. Since $J_{\mathrm{bit}}=0$ implies that $J_{\mathrm{cw}}=0$, the implemented search is lexicographic. Candidates satisfying $J_{\mathrm{bit}}\geq -\varepsilon$ for a small tolerance ε are preferred. Among these candidates, or, in their absence, among all candidates, $J_{\mathrm{cw}}$ is maximized, with $J_{\mathrm{bit}}$ serving as the final tie-breaker. Since the start margins depend only on the start pilots and the end margins only on the end pilots, the two searches decouple and are carried out exhaustively. For SP labeling, Theorem 1 yields $J_{\mathrm{bit}}=0$ in closed form. For Gray labeling, the search of Section 6.3 shows that the bit-level condition is attainable only for 4-PAM and 8-PAM, whereas the codeword-level condition $\Delta_{k}\geq 0$ is attained up to numerical precision for all constellation sizes considered.

### 6.2. SP Labeling: A Closed-Form Pilot Rule with Non-Negative Margins

For a scalar a>0 and a finite set Ω⊂R, let aΩ≜{aω:ω∈Ω}. For a sub-constellation Ω⊆X with average energy $E\left(\Omega \right)≜E_{X\in \Omega }\left[X^{2}\right]$ under a uniform input, so that $E\left(X_{t}\left(p_{t}\right)\right)=E_{t}\left(p_{t}\right)$, the unit-power re-normalization is denoted by

(28) $$N_{\mathrm{sub}}\left[\Omega \right]\;≜\;\Omega /\sqrt{E(\Omega )},$$

so that $X_{P}=N_{\mathrm{sub}}\left[X_{t}\left(p_{t}\right)\right]$ in (20) for $P=P_{t}\left(p_{t}\right)$.

For SP (natural binary) labeling of *M*-PAM with $M=2^{m}$, a symbol with label $b=(b_{0},\ldots ,b_{m-1})$, where $b_{0}$ is the MSB, admits the hypercube projection

(29) $$\mu \left(b\right)\;=\;\kappa \sum_{i=0}^{m-1}2^{\;m-1-i}\;\left(2b_{i}-1\right),\;\kappa =\sqrt{3/(M^{2}-1)},$$

where κ enforces $E[X^{2}]=1$.

**Lemma** **2** (Pilot-conditioned subsets are translations)**.** *Fix a pilot index subset I⊆{0,…,m−1} and pilot values $a={\left(a_{i}\right)}_{i\in I}$. Conditioned on $B_{i}=a_{i}$ for i∈I, the SP symbol in* (29) *decomposes as*

(30) $$X=\kappa \;\delta \left(a\right)+\kappa \;S,\;\delta \left(a\right)≜\;\;\sum_{i\in I}\;2^{\;m-1-i}\left(2a_{i}-1\right),\;\;S≜\;\;\sum_{i\notin I}\;2^{\;m-1-i}\left(2B_{i}-1\right),$$

*where S does not depend on a. The sub-constellations $X_{t}\left(a\right)$ for different a are therefore mutual translations, and their average energy is*

(31) $$E\left(X_{t}\left(a\right)\right)=\kappa^{2}\;\delta {\left(a\right)}^{2}+E_{s}^{\mathrm{rel}},\;E_{s}^{\mathrm{rel}}≜\kappa^{2}\;\;\sum_{i\notin I}\;4^{\;m-1-i}.$$

**Proof.**  The sum in (29) is split over I and its complement. Since the free bits are independent and uniformly distributed, E[S]=0 and $E\left[S^{2}\right]=\sum_{i\notin I}4^{m-1-i}$, which yields (31). □

**Lemma** **3** (Scale monotonicity of mutual information)**.** *Let X take values in a finite constellation* Φ *with a binary labeling, let $Z∼N(0,\sigma^{2})$ be independent of X, and for c>0, let $I^{\left(c\right)}\left(B;Y|\cdot \right)≜I\left(B;\;cX+Z|\cdot \right)$. Then, $I^{\left(c\right)}$ is nondecreasing in c under every conditioning on label bits.*

**Proof.**  Let $0<c_{1}<c_{2}$ and $\rho =c_{1}/c_{2}\in \left(0,1\right)$, and let $Z^{\prime }∼N\left(0,\sigma^{2}\left(1-\rho^{2}\right)\right)$ be independent of (B,X,Z). Since $\rho Z+Z^{\prime }∼N\left(0,\sigma^{2}\right)$, the observation $\rho \;\left(c_{2}X+Z\right)+Z^{\prime }$ has the same conditional law given (B,X) as $c_{1}X+Z$. Consequently, $B\to c_{2}X+Z\to c_{1}X+Z$ forms a Markov chain under every conditioning event, and the data-processing inequality yields $I^{\left(c_{1}\right)}\left(B;Y|\cdot \right)\leq I^{\left(c_{2}\right)}\left(B;Y|\cdot \right)$. □

By (28) and Lemma 2, the re-normalization of $X_{t}\left(a\right)$ to unit power removes the translation κδ(a) and multiplies *S* by the factor

(32) $$c^{⋆}\left(a\right)={\left(\kappa^{2}\delta {\left(a\right)}^{2}+E_{s}^{\mathrm{rel}}\right)}^{-1/2},$$

that is, $N_{\mathrm{sub}}\left[X_{t}\left(a\right)\right]=c^{⋆}\left(a\right)\;\kappa \;S$ up to translation, where $c^{⋆}\left(a\right)=1/\gamma_{P}$ for $P=P_{t}\left(a\right)$.

**Definition** **4** (Minimum-shift pilot)**.** *For a pilot set I, the minimum-shift pilot assignment is*

(33) $$a^{⋆}≜\underset{a\in {\left\{0,1\right\}}^{\left|I\right|}}{\arg\min}\left|\delta (a)\right|.$$

**Remark** **3** (Ties are harmless)**.** *The minimizer is never unique, since δ(1−a)=−δ(a). By* (32), *$c^{⋆}$ depends on a only through |δ(a)|, and S does not depend on a, so that all minimizers yield identical boundary rates and margins. The implementation selects one minimizer uniformly at random.*

**Theorem** **1** (Non-negative boundary rate margin under SP labeling)**.** *Consider M-PAM with $M=2^{m}$ and SP labeling, together with an arbitrary staircase delay vector d. Let the minimum-shift assignment of Definition 4 be applied to the active pilot positions $I_{t}$ at every boundary time t. Then, for every boundary pair $\left(k,i\right)\in D_{\mathrm{bd}}$ and every SNR,*

(34) $$\Delta_{k,i}\left(d,\{p_{t,i}^{⋆}\}\right)\;\geq \;0,$$

*so that every staircase delay vector is bit-level CMCA under SP labeling. Equality holds for a start-boundary pair with a single fixed bit at $t=\psi_{i}\left(k\right)$. For start pairs with two or more fixed bits and for all end pairs, the margin is strictly positive before the bit channel saturates.*

**Proof.**  The following is a sketch of the proof. Let $t=\psi_{i}\left(k\right)$. By Lemma 2, the middle-region term $C_{i}^{\mathrm{mid}}\left(d\right)$ equals $I^{\left(1\right)}\left(B_{t,i};Y_{t}|H_{t,i}\right)$, because averaging over the values of the side-information bits amounts to averaging over translations of the same free component *S*. The boundary term is the same functional evaluated on $N_{\mathrm{sub}}\left[X_{t}\left(a^{⋆}\right)\right]=c^{⋆}\kappa S_{\mathrm{bd}}$, where $S_{\mathrm{bd}}$ denotes the free sum over the levels that are neither fixed nor conditioned. The minimum-shift rule yields $\delta {\left(a^{⋆}\right)}^{2}\leq \sum_{i\in I_{t}}4^{m-1-i}$, which, in combination with (31) and $1=\kappa^{2}\sum_{i=0}^{m-1}4^{m-1-i}$, implies that $c^{⋆}\geq 1$. At a start time, Lemma 1 yields $I_{t}\cup H_{t,i}^{\mathrm{bd}}=\left\{j:d_{j}>d_{i}\right\}$, so that $S_{\mathrm{bd}}=S$, and Lemma 3, together with $c^{⋆}\geq 1$, establishes (34). At an end time, $S_{\mathrm{bd}}$ is obtained from *S* by additionally fixing the low-delay levels $I_{t}$. Fixing a level acts as conditioning up to a translation, which cannot reduce the mutual information, and Lemma 3 supplies the remaining step. The statements on equality and strict positivity follow from whether $\delta {\left(a^{⋆}\right)}^{2}$ attains $\sum_{i\in I_{t}}4^{m-1-i}$, which occurs exactly when $|I_{t}|=1$. The complete proof and an 8-PAM example are given in Appendix B. □

The proof relies on the labeling only through Lemma 2. Labelings without the hypercube property, such as Gray labeling, do not yield mutual translations under pilot fixing, and their margins may be negative even under the best pilots.

### 6.3. Gray Labeling: Pilot Design from Boundary Capacity

For Gray labeling, the pilots are obtained by the exhaustive search of Section 6.1, prior to and independently of any LDPC code design. The resulting assignments are held fixed in the density evolution and simulation studies that follow, and are tabulated in Appendix A for every delay vector used in this paper. Throughout the paper, the *worst-case* pilots denote the mirror image of this rule on the same search space, namely the assignment that minimizes $J_{\mathrm{cw}}$ with $J_{\mathrm{bit}}$ as the tie-breaker, and serve to expose the most severe bottleneck.

**Figure 6:**
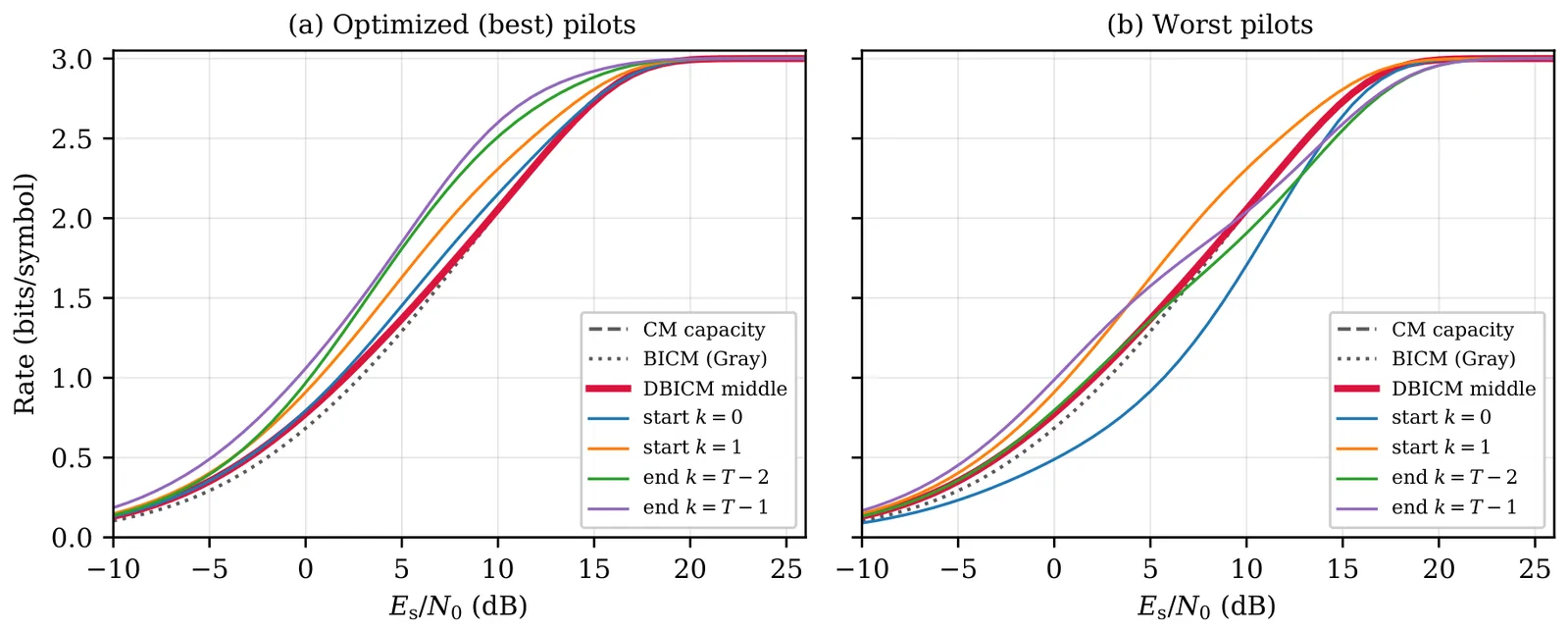
Per-codeword boundary rates for 8-PAM, Gray labeling, d=(1,0,2), for the boundary codewords k∈{0,1,T−2,T−1}, together with the middle-region capacity, the CM capacity, and the BICM rate. (**a**) Optimized pilots. (**b**) Worst-case pilots.

Figure 6 shows the per-codeword rates for 8-PAM with d=(1,0,2), which is the vector with the lowest DE threshold at R=1/2 (Section 7.3) and lies outside the family of Table 2 introduced below. Under the optimized pilots, every boundary codeword follows the middle-region capacity within $3\times 10^{-3}$ bits/symbols. Under the worst-case pilots, codeword k=0 falls below it by up to 0.46 bits/symbols. The discrepancy can be traced to two pilot bits. At t=0, the levels with $d_{j}>0$, namely $\left(B_{0},B_{2}\right)$, are fixed, and only $B_{1}$ carries information. The optimized setting $\left(B_{0},B_{2}\right)=\left(0,0\right)$ leaves the pair $\left\{-7,-1\right\}/\sqrt{21}$, whose re-normalized spacing is $6/\sqrt{25}=1.20$. The worst-case setting (0,1) leaves the adjacent pair $\left\{-5,-3\right\}/\sqrt{21}$ with a spacing of $2/\sqrt{17}=0.49$, which amounts to a penalty of 7.9 dB on this single bit channel. At the end times, the level-1 pilot selects either the inner points $\left\{\pm 1,\pm 3\right\}/\sqrt{21}$, whose spacing is expanded from 0.44 to 0.89 by the unit-power re-normalization, or the outer points $\left\{\pm 5,\pm 7\right\}/\sqrt{21}$, whose spacing is compressed to 0.33. Favorable pilots are therefore those whose sub-constellations are re-normalized into widely spaced points, and unfavorable patterns of this kind exist for every constellation and delay vector studied here.

**Remark** **4** (Boundary rates may exceed the CM capacity)**.** *In Figure 6, the end-boundary codewords exceed the CM capacity at a low SNR, and even the Gaussian capacity $\frac{1}{2}\log_{2}\left(1+1/\sigma^{2}\right)$, with 0.1861 versus 0.1315 bits/symbols at $E_{s}/N_{0}=-10$ dB. This does not violate the converse. The m summands of $C_{\mathrm{DBICM}}\left(k;\cdot \right)$ are located at m distinct symbol times. For a middle-region codeword, the nested conditioning of *(12)* telescopes to a single term I(X;Y), so that the codeword receives exactly the capacity of one symbol. A boundary codeword, by contrast, shares its symbol times with pilots, which carry no information while the surviving points are re-normalized to full power. The telescoping is thereby broken, and the sum may exceed the capacity of one symbol. The frame rate $\frac{1}{T_{s}}\left(|K_{\mathrm{mid}}|C_{\mathrm{DBICM}}^{\mathrm{mid}}+\sum_{k\in K_{\mathrm{bd}}}C_{\mathrm{DBICM}}\left(k;\cdot \right)\right)$, which counts one channel use per symbol time, stays below the Gaussian capacity for every T (0.1231 versus 0.1315 at −10 dB for T=20) and converges to $C_{\mathrm{DBICM}}^{\mathrm{mid}}\left(d\right)$ as T→∞. The margin $\Delta_{k}$ is consequently a relative measure with respect to the middle region, and the binding constraints of the design arise at the start boundary.*

Table 2 lists, for each constellation, the Gray-labeled delay vectors whose optimized pilots keep every boundary codeword at or above the middle-region capacity, together with the deepest per-codeword margin $\min_{k,\mathrm{SNR}}\Delta_{k}$ and the deepest per-bit margin $\min_{k,i,\mathrm{SNR}}\Delta_{k,i}$. For 4-PAM and 8-PAM, the listed vectors exhibit no negative residual in either quantity to the eight decimal digits of the computation, and are therefore bit-level CMCA to numerical precision. For 16-PAM and 32-PAM, no delay vector is bit-level CMCA. Individual per-bit margins reach −0.23 to −0.32 bits/symbols, because at a start time, the pilots occupy positions that would otherwise supply decoded side information to the target bit, and Gray labeling offers no translation structure that could compensate for this loss within the same bit level. The loss is nevertheless recovered within the codeword by the remaining levels, which benefit from the re-normalization of the pilot-restricted sub-constellation. The deepest per-codeword deficits amount to only $8\times 10^{-7}$ bits/symbols for 16-PAM and $1\times 10^{-8}$ bits/symbols for 32-PAM. They occur exclusively in the high-SNR saturation regime and lie within the resolution of the numerical quadrature, for which three independent integration routines agree to $5\times 10^{-7}$. These vectors are therefore CMCA in the codeword-level sense of Definition 1 to numerical precision. A delay vector is termed *nearly best* if its optimized pilots yield $\Delta_{k}\geq -\varepsilon$ for all boundary codewords and all grid SNRs, where $\varepsilon =10^{-6}$ bits/symbols. The family comprises 2 of the 24 staircase vectors for 16-PAM and 9 of the 120 staircase vectors for 32-PAM. The closest excluded vectors have deepest per-codeword margins of $-3.3\times 10^{-4}$ bits/symbols for 16-PAM and $-3.8\times 10^{-6}$ bits/symbols for 32-PAM. Under SP labeling, no staircase vector exhibits a negative per-bit residual, in agreement with Theorem 1.

## 7. Density Evolution

### 7.1. Position-Dependent Channels and System Threshold

Density evolution (DE) for DBICM differs from the binary-input AWGN setting [27,28] in four respects. First, the bit-level LLR distributions of a PAM constellation are non-Gaussian, so that the initial densities $c_{i}\left(\cdot \right)$ are estimated by Monte Carlo simulation and tracked on a fine LLR grid [29,30]. Second, since the coded bits are demultiplexed onto the *m* levels as in BICM [5,6], a variable node observes the mixture $\sum_{i}w_{i}c_{i}$, where $w_{i}$ is the fraction of variable nodes assigned to level *i*, equal to 1/m for the regular codes used here. Third, SP labeling renders the channel asymmetric, so that the LLR densities $P_{0}$ and $P_{1}$ conditioned on the transmitted bit being 0 and 1 are tracked separately and symmetrized before the check-node update by the coset-code argument, which yields the symmetric density $v_{s}\left(L\right)=\frac{1}{2}\left(P_{0}\left(L\right)+P_{1}\left(-L\right)\right)$ [8,30]. Fourth, the bits sharing a symbol belong to different codewords. At a DE fixed point, the messages of a successfully decoded codeword concentrate at ±∞, so that already-decoded bits act as perfectly known side information, and the target bit channel is obtained by averaging over their equiprobable values. This coset marginalization reproduces the conditioning of Section 5 exactly, because the conditional mutual information is defined by the same average. Undecoded co-located bits are marginalized under uniform priors, whereas pilot bits are fixed, which restricts the constellation to a sub-constellation re-normalized by $1/\gamma_{P}$. The channel is therefore position dependent, with pilot-conditioned mixtures at the boundary codewords and a stationary mixture in the middle region. The finite-length receiver of Section 8, by contrast, conditions on the finite a posteriori LLRs of the decoder, which are sample realizations rather than point masses.

Decoding proceeds codeword by codeword, so that the block is decoded if and only if DE converges at every position given its already-converged predecessors. This genie-aided condition is exact within the DE model but optimistic for SP labeling at finite length, where a decoding error propagates through the feedback path (Section 8.1.4). Let $\theta_{\mathrm{pos}}$ denote the convergence threshold of the mixture channel at a given position. Since success requires convergence at every position and thresholds are measured in SNRs, the system threshold is the maximum

(35) $$\theta ≜\max_{\mathrm{pos}}\theta_{\mathrm{pos}}$$

over the $d_{\max}$ start positions, the middle region, and the $d_{\max}$ end positions. The frame length *T* does not enter the recursion, since additional codewords merely replicate middle-region positions, and it affects the comparison only through $R_{\mathrm{eff}}$ in (17). The initial densities are estimated from $10^{7}$ channel realizations per level, position, and SNR, and from $10^{8}$ realizations in the high-sample runs that are used in Section 7.3 wherever two candidates differ by less than 0.01 dB. A position is declared convergent when its error probability falls below $10^{-6}$ within 100 iterations, and $\theta_{\mathrm{pos}}$ is determined by bisection with common random numbers.

### 7.2. Rate Matching and Thresholds with the Designed Pilots

A DBICM frame occupies $T_{s}=T+d_{\max}$ symbol times for *T* codewords, so that its effective rate $R_{\mathrm{eff}}$ in (17) is smaller than *R*. The DE thresholds are independent of *T*. In the finite-length simulations, however, the overhead is transmitted, and a comparison with BICM at the nominal rate would favor DBICM. The BICM baseline is therefore matched to $R_{\mathrm{eff}}$ by shortening the mother code, that is, by fixing $sn_{c}$ information bits, which constitute a fraction *s* of the code length $n_{c}$, to known values. This yields the transmitted rate $R^{\prime }=\left(R-s\right)/\left(1-s\right)$ on the same Tanner graph. With $T=249\;d_{\max}$, the effective rate is $R_{\mathrm{eff}}=0.996R$, which is matched by s=0.0040 ($R^{\prime }=0.4980$) at R=1/2 and by s=0.0013 ($R^{\prime }=0.2490$) at R=1/4. In DE, a shortened bit is a variable node with a perfectly reliable observation. Shortening consequently improves the baseline $E_{s}/N_{0}$ threshold by at most 0.05 dB and the $E_{b}/N_{0}$ threshold at $R^{\prime }$ by at most 0.034 dB (Table 3). For the regular ensembles used here, all variable nodes have the same degree, so that the shortened positions do not affect the threshold, and their selection is of consequence only at finite length (Section 8.1.1). Shortening thus favors the baseline slightly, and the gains reported below are conservative.

All evaluations employ the regular (3,6) ensemble at R=1/2 and the regular (3,4) ensemble at R=1/4, which isolate the effects of the delay vector, the labeling, and the pilots and yield reproducible thresholds. For each constellation, labeling, and rate, Table 4 reports the best staircase delay vector under the designed pilots, where $E_{b}/N_{0}=E_{s}/N_{0}-10\log_{10}\left(mR_{\mathrm{eff}}\right)$. Since both systems are rate matched and share this offset, the gain is identical on the $E_{s}/N_{0}$ and $E_{b}/N_{0}$ scales. Two conventions are presented. The finite-*T* regime employs $R_{\mathrm{eff}}=0.996R$ and the shortened baseline, in accordance with the simulations of Section 8. The asymptotic regime T→∞ employs $R_{\mathrm{eff}}=R$ and the unshortened baseline, which constitutes the information-theoretic benchmark. The $E_{s}/N_{0}$ column is common to both regimes. Relative to the finite-*T* column, the DBICM $E_{b}/N_{0}$ in the asymptotic column is lower by $10\log_{10}\left(R/0.996R\right)=0.017$ dB, whereas the baseline forfeits the $E_{b}/N_{0}$ advantage conferred by shortening, which is at most 0.034 dB (Table 3, 32-PAM at R=1/2). The two “Gain” columns of Table 4 therefore differ by the sum of these two quantities, between 0.01 and 0.05 dB after rounding.

### 7.3. Observations

Four observations follow from Table 3 and Table 4, concerning the size of the gain, the role of the labeling, the choice of the delay vector at a fixed rate, and the structure of the best delay vectors.

**The gain grows with the constellation size and with decreasing rate.** In the finite-*T* regime, the gain over the rate-matched baseline increases from 0.06 to 0.09 dB for 4-PAM at R=1/2 to 1.02 to 1.03 dB for 32-PAM at R=1/4. In the asymptotic regime, it increases from 0.09 to 1.06 dB.

**SP labeling matches Gray labeling under DE.** For every (M,R), the best Gray and SP thresholds agree within a few hundredths of a decibel, and within a few thousandths for 16-PAM and 32-PAM. This agreement is remarkable, because under BICM the two labelings differ substantially; for instance, 11.916 dB for Gray labeling versus 15.373 dB for SP labeling with unshortened 16-PAM at R=1/2. The delay yields 4.0 dB for SP labeling, which more than recovers this penalty, whereas it yields 0.55 dB for Gray labeling. The underlying mechanism is Proposition 1, according to which every staircase vector attains I(X;Y) in the middle region for an arbitrary labeling, and the thresholds of a fixed ensemble reflect this equivalence. The SP baseline is omitted from the gain columns, because its BICM loss would inflate the reported gains. The equivalence presupposes error-free side information and does not survive at finite length, where SP labeling suffers from error propagation (Section 8.1.4).

**The threshold-optimal delay vector need not belong to the capacity-preserving family.** The winning vectors are staircase permutations, whereas membership in the capacity-preserving family of Table 2 is incidental. For 8-PAM with Gray labeling, Table 4 selects (2,0,1) at R=1/4, which belongs to the family, and (1,0,2) at R=1/2, which is not a member of the family. The two tables answer different questions. Table 2 concerns the safety of the boundary, whereas Table 4 concerns the threshold of one ensemble at one rate, which is dominated by the middle region. Under optimized pilots, the deepest per-codeword deficit of (1,0,2) is only $3\times 10^{-3}$ bits/symbols, and Table 5 reports 7.520 dB for (1,0,2) against 7.522 dB for the capacity-preserving vector (0,2,1). The bit-level CMCA property thus constitutes a safety margin rather than a threshold gain, and its value becomes apparent under poor pilots (Section 7.4). Moreover, the choice is robust, since 15 of the 24 vectors lie within 0.05 dB of the optimum for 16-PAM with Gray labeling at R=1/2, and 65 of the 120 vectors for 32-PAM, while the spread among the five leading vectors narrows from 0.03 to 0.004 dB.

**The delay vector determines the decoding order, and interleaving permutations attain the lowest thresholds.** Since level *i* is conditioned on $\left\{j:d_{j}>d_{i}\right\}$, the vector d specifies the successive decoding order, from the largest delay, which receives no side information, to $d_{i}=0$, which receives all of it. All m! staircase permutations are capacity equivalent by Proposition 1, so that the ranking is determined by the shape of the LLR mixture alone. This explains the small spread and its sensitivity to numerical precision. For 16-PAM with Gray labeling at R=1/2, for instance, (0,1,2,3) leads (2,1,0,3) by 0.003 dB in the standard runs and trails it by 0.001 dB in the high-sample runs. What persists is a tendency toward interleaving. For 4-PAM and 8-PAM, every winner and every member of Table 2 is an alternating permutation, and for 16-PAM and 32-PAM, the leading vectors still alternate between large and small delays, typically with the largest delay on the LSB and zero delay on the second least significant level.

### 7.4. Worst-Case Pilot Thresholds

Table 5 re-evaluates the 8-PAM thresholds under the worst-case pilots of Section 6.3, which are listed in Appendix A. Under Gray labeling, the penalty ranges from 1.47 dB for (0,2,1) at R=1/2 to 3.66 dB for (1,0,2) at R=1/4, because the boundary rates fall below the middle-region rate (Figure 6b) and become bottlenecks in (35). SP labeling loses at most 0.75 dB, and only 0.05 dB for (0,1,2) at R=1/2, because, by Lemma 2, no pilot assignment can collapse the boundary distances. Since the worst-case pilots are selected by the mutual-information criterion rather than by the DE threshold, these penalties constitute lower bounds on the loss caused by poor pilots. Finite-length simulations in the setting of Section 8.1.1 confirm the location of the failure. For (0,2,1), the start-region BER is 0 and the end-region BER is $6.3\times 10^{-2}$ at $E_{s}/N_{0}=7.75$ dB. For (1,0,2), the start region dominates with $8.4\times 10^{-2}$ against $4.6\times 10^{-2}$ in the end region, in agreement with the deficit of −0.46 bits/symbols at k=0 in Figure 6b.

## 8. Simulation

This section validates the DE analysis by Monte Carlo simulation, first with the regular (3,6) and (3,4) codes and subsequently with irregular codes.

### 8.1. Regular LDPC Codes

#### 8.1.1. Setup

The simulations employ the designed pilots of Section 6.3 and the delay vectors of Table 4, Table 5 and Table A2, with T=249 for $d_{\max}=1$ and T=498 for $d_{\max}=2$. The parity-check matrices have length $n_{c}=60,000$ and are constructed by progressive edge growth with ACE constraints [31,32]. The same matrix is reused for all delay vectors, labelings, and pilots at a given rate. The demapper computes exact bit LLRs [5] without the max-log approximation [6]. It marginalizes the undecided co-located bits under uniform priors and conditions on the finite a posteriori LLRs of previously decoded codewords, so that the delayed feedback is soft. Decoding is performed by log-domain belief propagation [33] with at most 100 iterations per codeword. Each DBICM point aggregates up to $10^{6}$ transmissions of *T* codewords each and is terminated after 500 codeword errors, whereas each BICM point aggregates up to $4\times 10^{7}$ codewords and is terminated after 100 codeword errors.

The BICM baseline is the same mother code, shortened as in Section 7.2. At the transmitter, the last $sn_{c}$ positions of the codeword in the column order of the parity-check matrix are set to zero and are not transmitted, namely 240 bits at R=1/2 and 78 bits at R=1/4, which correspond to whole symbols for both constellations and yield $R^{\prime }=0.4980$ and $R^{\prime }=0.2490$, as in Section 7.2. Since the parity-check matrix is itself a random construction, no separate interleaver is employed, and the position of the shortened block is immaterial. At the receiver, the corresponding variable nodes are initialized with a large positive LLR of +50, which represents a certain zero within the numerical range of the decoder. The shortened bits therefore supply certain messages to their check nodes and can only benefit the baseline.

#### 8.1.2. Effect of the Pilot Values

**Figure 7:**
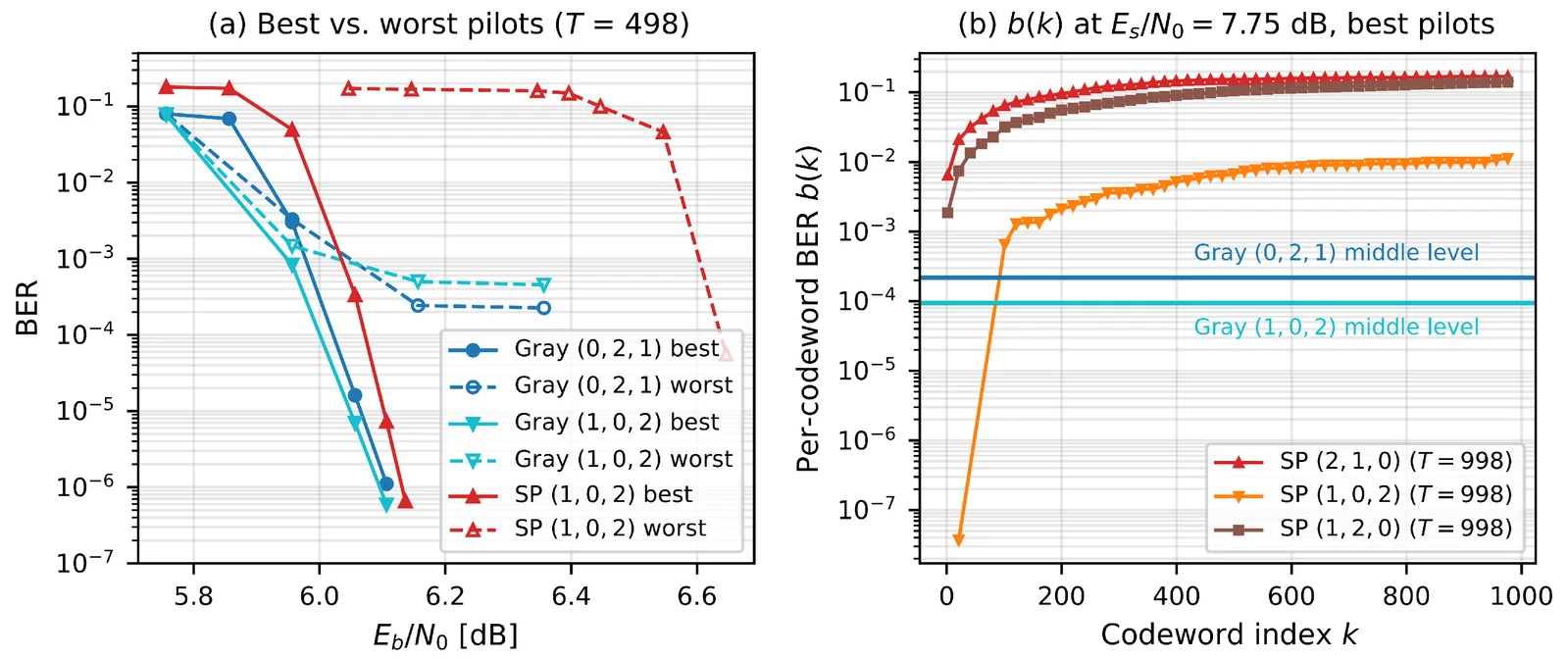
8-PAM at R=1/2. (**a**) Designed versus worst-case pilots (T=498). (**b**) Per-codeword BER b(k) at $E_{s}/N_{0}=7.75$ dB under designed pilots (T=998); horizontal lines mark the middle-region level for Gray labeling.

Figure 7a shows 8-PAM at R=1/2 with T=498. Under the worst-case pilots, the curves are not merely shifted but develop an early error floor for both labelings, since the boundary codewords fail irrespective of the SNR margin of the middle region. Under the designed pilots, the same delay vectors exhibit clean waterfalls, in agreement with Table 5 and Figure 6b. All subsequent simulations employ the designed pilots.

#### 8.1.3. Waterfalls Against the BICM Baseline

**Figure 8:**
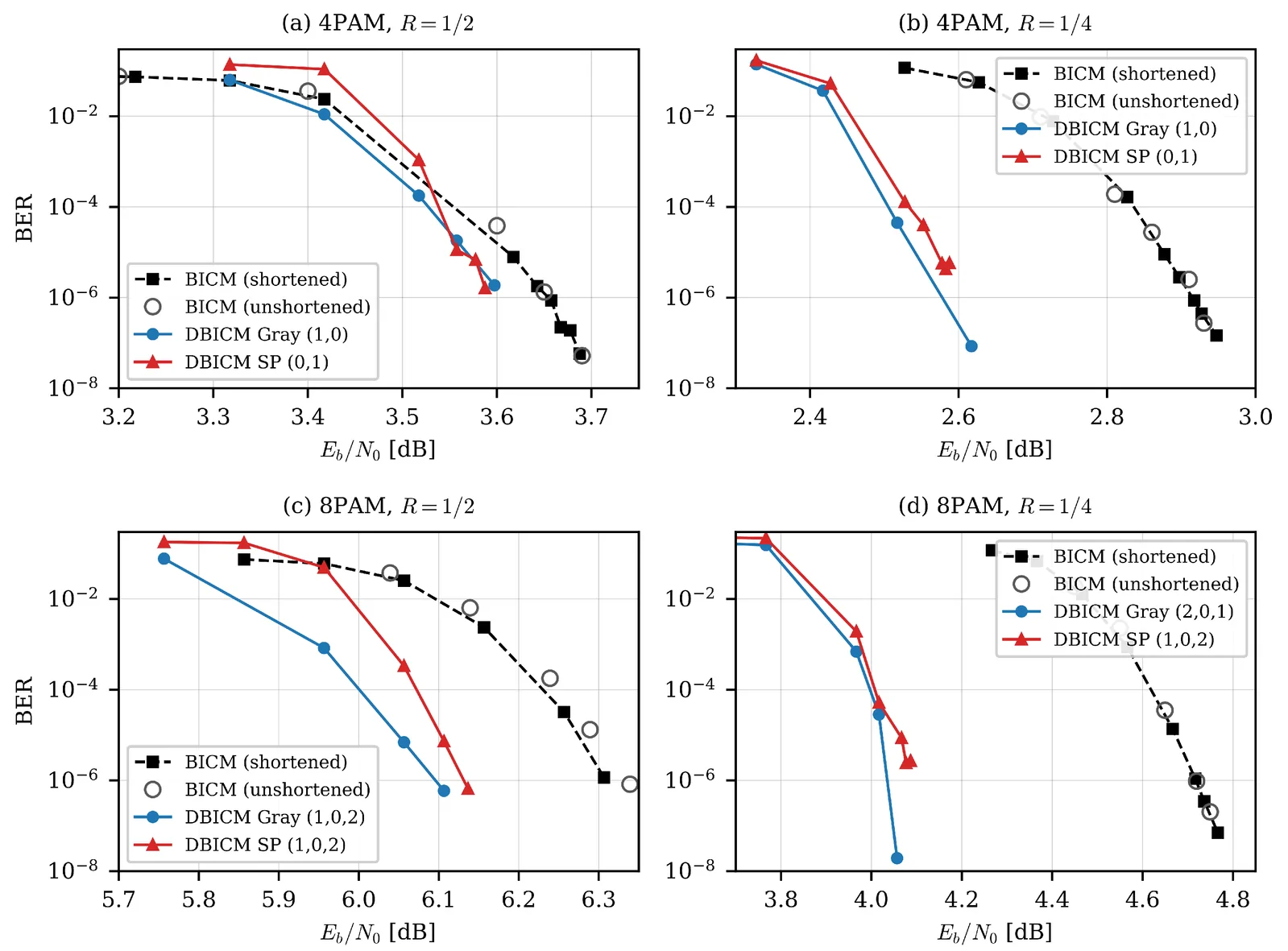
BER versus $E_{b}/N_{0}$ for DBICM with designed pilots (T=249 for $d_{\max}=1$, T=498 for $d_{\max}=2$), the BICM baseline shortened to the same effective rate, and the unshortened mother code at its nominal rate. (**a**) 4-PAM, R=1/2. (**b**) 4-PAM, R=1/4. (**c**) 8-PAM, R=1/2. (**d**) 8-PAM, R=1/4.

Figure 8 shows the BER for 4-PAM and 8-PAM at both rates. To verify that the shortening does not distort the comparison, the unshortened mother code, transmitted at its nominal rate and plotted with $R_{\mathrm{eff}}=R$, is included in Figure 8. The shortened and unshortened baselines coincide within 0.02 dB at $\mathrm{BER}=10^{-5}$ in all four panels, so that neither the amount nor the position of the shortening affects the reported gains. At $\mathrm{BER}=10^{-5}$, the gains over the shortened baseline for Gray and SP labeling are 0.04 and 0.05 dB in panel (a), 0.33 and 0.31 dB in panel (b), 0.23 and 0.17 dB in panel (c), and 0.65 and 0.61 dB in panel (d), respectively. For Gray labeling, these values follow the finite-*T* predictions of Table 4 to within approximately 0.1 dB. The SP curves deviate in two respects. Their gains fall short of the DE predictions, as in panel (c), where Gray labeling lies within 0.03 dB of the prediction while SP labeling falls 0.11 dB short. At a low BER, they are irregular, with a floor near $5\times 10^{-6}$ in panel (b) and non-monotone final points in panel (d). A region-resolved error audit attributes this floor entirely to the middle region, with no errors at either boundary, which excludes both the pilots and the shortening of the baseline as its cause. The floor is caused by inter-codeword error propagation, which is quantified next. The gray curves exhibit steep waterfalls down to $\mathrm{BER}<10^{-7}$ and do not degrade as *T* increases. Below $\mathrm{BER}=10^{-6}$, the slope of the BICM curves of the (3,4) code at R=1/4 decreases gradually, to about a factor of two per 0.01 dB, and the curves reach $1.5\times 10^{-7}$ for 4-PAM and $7\times 10^{-8}$ for 8-PAM at the deepest simulated points, which lie two orders of magnitude below the level at which the gains are measured. Each failing frame in this region contains several thousand erroneous bits, so that the failures are undecoded frames rather than absorbing-set events. The behavior is thus a finite-length property of the (3,4) code near its threshold and is unrelated to the shortening, because the shortened and unshortened baselines coincide over the depth reached by both.

#### 8.1.4. Labeling and Error Propagation

Let b(k) denote the BER of the *k*-th codeword of a frame, averaged over frames. Figure 7b shows b(k) under the designed pilots at T=998, where the frame length is doubled in order to expose the accumulation of errors along the frame. For Gray labeling, b(k) is independent of the position, with no boundary errors at this SNR, and coincides with the middle-region level indicated by the horizontal lines. For SP labeling, b(k) increases monotonically for all three delay vectors shown, namely (2,1,0), (1,0,2), and (1,2,0), so that the effect is intrinsic to the labeling. Under Gray labeling, adjacent points differ in a single bit, so that a nearest-neighbor error corrupts one level. Under SP labeling, adjacent points differ in several bits, and in all *m* bits at the center of the constellation, where 011⋯1 meets 100⋯0, so that a single symbol error injects erroneous side information into several codewords simultaneously. At $E_{s}/N_{0}=7.75$ dB for 8-PAM, R=1/2, and d=(2,1,0), b(k) rises from $6.5\times 10^{-3}$ at k=2 to $1.2\times 10^{-1}$ at k=280, and saturates near $1.5\times 10^{-1}$. The frame-averaged performance of SP labeling therefore deteriorates with *T* and never reaches its DE threshold, whereas Gray labeling attains it. DE cannot capture this effect, because it presupposes error-free side information. The propagation occurs although the feedback is already soft, and hard-decision feedback would only aggravate it. A similar phenomenon has been reported for convolutional polar coded modulation with SP labeling, where an ad hoc backward decoding pass serves as a remedy (Section IV-A in [22]). The present paper instead removes the cause by adopting Gray labeling.

### 8.2. Irregular LDPC Codes

It remains to examine whether the gain survives the optimization of the degree distribution. The codes have one variable-node degree profile per bit level, with degrees in {2,3,4,8,10}, a check-node degree of 7, $n_{c}=60,000$, and R=1/2, and the profiles are optimized for BICM. Gray labeling and the designed pilots are employed throughout, and the BICM baseline is shortened by 240 bits exactly, as in Section 8.1.1. Table 6 lists the DE thresholds, and Table 7 lists the profiles and their assignment.

**Figure 9:**
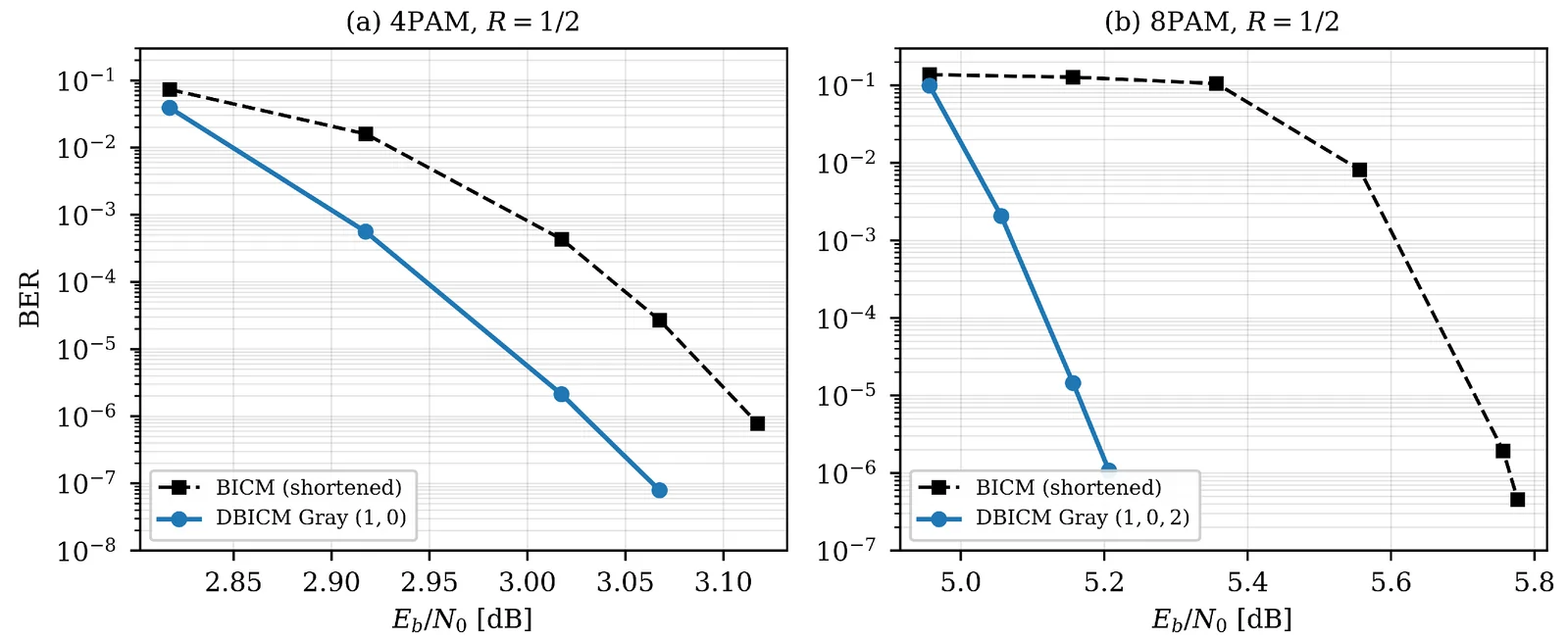
Irregular LDPC codes (degrees in {2,3,4,8,10}, check degree 7) under Gray labeling at $R_{\mathrm{eff}}=0.498$ with designed pilots. (**a**) 4-PAM, d=(1,0), T=249, same code under BICM and DBICM. (**b**) 8-PAM, d=(1,0,2), T=498, shared profiles with permuted assignment.

For 4-PAM with d=(1,0), the BICM-optimized code serves DBICM without modification. At m=2, the delay alters the bit channels only mildly, since one level gains side information while the other remains the marginalized BICM channel, so that the per-level disparity to be absorbed by the profile is similar in both systems. The DE gain at $R_{\mathrm{eff}}=0.498$ is 0.098 dB, and Figure 9a shows a gain of 0.09 dB at $\mathrm{BER}=10^{-5}$ for the same code under both systems with T=249.

For 8-PAM with d=(1,0,2), the two systems share the three profiles *A*, *B*, and *C* of Table 7, whose class-average degrees are 2.5, 4.0, and 4.0 and of which only *C* contains degree-10 nodes, and they differ solely in the assignment of the profiles to the levels. BICM employs the assignment (A,B,C), whereas DBICM employs (B,A,C), which places the lightest profile *A* on the zero-delay level, decoded last with full side information, and the heavier profiles on the levels decoded earlier. The DE gain is 0.520 dB, and Figure 9b shows a gain of 0.55 dB at a $\mathrm{BER}=10^{-5}$ with T=498.

Both gains are smaller than those of the regular codes, since the irregular optimization and the delayed feedback exploit the same asymmetry among the bit channels. The overlap is nevertheless partial, and the gain persists. Adapting the code to the delay is inexpensive. No change is required for m=2, and only a permutation of the level assignment is required for m=3, so that the profile follows the decoding order. A systematic treatment of this assignment problem for m≥4 is left for future work.

## 9. Conclusions

This paper has analyzed DBICM as a continuous transmission of a finite train of *T* codewords, in which the delays necessarily place pilot bits in the symbols at both ends of the frame. The achievable rate of every codeword in the start, middle, and end regions was formulated, and the boundary rates were shown to depend on the pilot values through the restriction of the sub-constellation and its unit-power re-normalization. The delay vector and the pilots were then designed such that no boundary codeword falls below the middle-region CM capacity. For SP labeling, the closed-form minimum-shift pilot rule guarantees this property at the bit level for every staircase delay vector and every SNR. For Gray labeling, which admits no such structure, exhaustive search yields pilots that preserve the capacity at the codeword level up to numerical precision for 4-PAM through 32-PAM.

A position-dependent density evolution analysis showed that, under the designed pilots, the boundary codewords are not bottlenecks and the thresholds of Gray and SP labeling coincide. The gain over rate-matched BICM grows with the constellation size and with decreasing rate, reaching approximately 1.05 dB for 32-PAM at a rate of 1/4, whereas poor pilots cost up to 3.7 dB under Gray labeling. Finite-length simulations confirmed the predictions for Gray labeling, with waterfalls at the DE thresholds and a performance invariant to *T*. For SP labeling, they revealed an inter-codeword error propagation that is invisible to both the capacity and the DE analyses, under which the per-codeword error rate grows along the frame and the performance deteriorates continuously with *T*. The recommended configuration is therefore Gray labeling combined with a staircase delay vector and capacity-designed pilots. With irregular LDPC codes, the gain persists and amounts to 0.09 dB for 4-PAM and 0.55 dB for 8-PAM, and the code follows the decoding order at negligible cost, without modification for 4-PAM and with a permuted level assignment for 8-PAM. The systematization of this assignment for larger constellations and the extension of the boundary analysis to multi-dimensional constellations are directions for future work.

## Figures and Tables

**Table 1:** Notation for pilots, boundary regions, and pilot-restricted constellations. Throughout, $t=\psi_{i}\left(k\right)$ denotes the symbol time at which the level-*i* bit of codeword *k* is transmitted, and P stands for the constraint $P_{t}\left(p_{t}\right)$ at that time.

Symbol	Definition	Meaning
$\psi_{i}\left(k\right)$	$k+d_{i}$	Integer. Symbol time at which the level-*i* bit of codeword *k* is transmitted, defined in (9).
$ineI_{t}$	$\left\{\;j:\;t-d_{j}\notin \left\{0,\ldots ,T-1\right\}\;\right\}$	Set of level indices j∈{0,…,m−1} whose position at time *t* is occupied by a pilot, defined in (10). Empty in the middle region.
$inep_{t}$	${\left(p_{t,j}\right)}_{j\in I_{t}}\in {\left\{0,1\right\}}^{|I_{t}|}$	Binary vector of the pilot values at time *t*, with one component $p_{t,j}$ for each index $j\in I_{t}$.
$ineP_{t}\left(p_{t}\right)$	$\left\{\;B_{t,j}=p_{t,j}:\;j\in I_{t}\;\right\}$	Set of equality constraints on the label bits of the symbol at time *t*, defined in (18).
$ineP_{k,i}$	$P_{\psi_{i}\left(k\right)}\left(p_{\psi_{i}\left(k\right)}\right)$	The constraint set experienced by the pair (k,i), namely $P_{t}\left(p_{t}\right)$ at $t=\psi_{i}\left(k\right)$, defined in (19).
$ineK_{\mathrm{start}}$	$\left\{0,\ldots ,d_{\max}-1\right\}$	Set of codeword indices *k* in the start region.
$ineK_{\mathrm{mid}}$	$\left\{d_{\max},\ldots ,T-d_{\max}-1\right\}$	Set of codeword indices *k* in the middle region, none of whose *m* symbol times carries a pilot.
$ineK_{\mathrm{end}}$	$\left\{T-d_{\max},\ldots ,T-1\right\}$	Set of codeword indices *k* in the end region.
$ineK_{\mathrm{bd}}$	$K_{\mathrm{start}}\cup K_{\mathrm{end}}$	Set of boundary codeword indices, defined in (23).
$ineD_{\mathrm{bd}}$	$K_{\mathrm{bd}}\times \left\{0,\ldots ,m-1\right\}$	Set of boundary index pairs (k,i), defined in (23).
$ineX_{t}\left(p_{t}\right)$	$\left\{\;\mu \left(b\right)\in X:\;b_{j}=p_{t,j},\;j\in I_{t}\;\right\}$	Subset of constellation points (raw sub-constellation) whose labels agree with the pilots at time *t*.
$ineE_{t}\left(p_{t}\right)$	$E\left[X^{2}\right],\;X∼\mathrm{Unif}\left(X_{t}\left(p_{t}\right)\right)$	Scalar. Average energy of the raw sub-constellation.
$ine\gamma_{P}$	$\sqrt{E_{t}\left(p_{t}\right)}$	Scalar. Root-mean-square amplitude used for re-normalization, with $\gamma_{\emptyset }=1$.
$ineX_{P}$	$\left\{\;x/\gamma_{P}:\;x\in X_{t}\left(p_{t}\right)\;\right\}$	Subset of the real line (re-normalized sub-constellation) with unit average power, defined in (20), with $X_{\emptyset }=X$.
$ineI_{X_{P}}\left(\;\cdot \;;\cdot |\cdot \right)$	—	Mutual information evaluated with the channel input uniformly distributed on $X_{P}$. The subscript encodes pilot fixing, and the conditioning bar encodes conditioning on random variables.
$ineH_{t,i}\left(d\right)$	$\left\{\;B_{t,j}:\;d_{j}>d_{i}\;\right\}$	Set of label-bit random variables of the symbol at time *t* that serve as side information for level *i* in the middle region, defined in (11).
$ineH_{t,i}^{\mathrm{bd}}\left(d\right)$	$H_{t,i}\left(d\right)\setminus \left\{\;B_{t,j}:\;j\in I_{t}\;\right\}$	Set of label-bit random variables serving as side information for level *i* in the boundary regions, obtained by removing the pilot positions, defined in (21).

**Table 2:** Gray-labeled delay vectors admitting capacity-preserving boundary pilots (nearly-best family, $\varepsilon =10^{-6}$ bits/symbols). Deepest per-codeword margin $\min_{k,\mathrm{SNR}}\Delta_{k}$ and deepest per-bit margin $\min_{k,i,\mathrm{SNR}}\Delta_{k,i}$ in bits/symbols, where 0 indicates no negative residual to eight decimal digits.

Constellation	Delay Vectors d	Family Size	Deepest $\Delta_{k}$	Deepest $\Delta_{k,i}$
4-PAM	(0,1), (1,0)	2/2	0	0
8-PAM	(0,2,1), (2,0,1)	2/6	0	0
16-PAM	(0,2,3,1), (3,1,0,2)	2/24	$-8\times 10^{-7}$	−0.28, −0.23
32-PAM	(0,2,3,4,1), (0,3,2,4,1), (1,2,3,4,0),	9/120	$-1\times 10^{-8}$	−0.26 to −0.32
	(1,3,2,0,4), (2,3,1,0,4), (3,2,1,0,4),			
	(4,1,2,0,3), (4,2,1,0,3), (4,3,1,0,2)			

**Table 3:** BICM baseline with code shortening: shortening fraction *s*, transmitted code rate $R^{\prime }=\left(R-s\right)/\left(1-s\right)$, and asymptotic DE thresholds (in dB).

		R=1/2			R=1/4		
		s	$E_{s}/N_{0}$	$E_{b}/N_{0}$	s	$E_{s}/N_{0}$	$E_{b}/N_{0}$
4-PAM	Original	0	3.400	3.400	0	−0.411	2.599
	Shortened	0.0040	3.372	3.390	0.0013	−0.428	2.599
8-PAM	Original	0	7.801	6.040	0	3.100	4.349
	Shortened	0.0040	7.766	6.022	0.0013	3.079	4.346
16-PAM	Original	0	11.916	8.906	0	6.036	6.036
	Shortened	0.0040	11.874	8.881	0.0013	6.010	6.028
32-PAM	Original	0	15.848	11.869	0	8.682	7.713
	Shortened	0.0040	15.797	11.835	0.0013	8.652	7.700

**Table 4:** DBICM DE thresholds with capacity-designed pilots and corresponding gains over the BICM baseline (Table 3) under the two rate conventions. *Finite T*: $T=249\;d_{\max}$, $R_{\mathrm{eff}}=0.996R$, benchmarked against the rate-matched shortened baseline. *Asymptotic* T→∞: $R_{\mathrm{eff}}\to R$, benchmarked against the original unshortened mother code baseline. The $E_{s}/N_{0}$ thresholds are identical for both regimes because DE convergence is independent of *T*, and only the $E_{b}/N_{0}$ conversion differs. $E_{b}/N_{0}^{\left(B\right)}$ denotes the BICM baseline threshold. All thresholds and gains are in dB.

	Label	d	$d_{\max}$	$E_{s}/N_{0}$	Finite *T* ($R_{\mathrm{eff}}=0.996R$)			T→∞ ($R_{\mathrm{eff}}=R$)		
					$E_{b}/N_{0}$	$E_{b}/N_{0}^{\left(B\right)}$	Gain	$E_{b}/N_{0}$	$E_{b}/N_{0}^{\left(B\right)}$	Gain
R=1/2										
4-PAM	Gray	(1,0)	1	3.313	3.331	3.390	0.06	3.313	3.400	0.09
4-PAM	SP	(0,1)	1	3.283	3.300	3.390	0.09	3.283	3.400	0.12
8-PAM	Gray	(1,0,2)	2	7.520	5.776	6.022	0.25	5.759	6.040	0.28
8-PAM	SP	(1,0,2)	2	7.490	5.746	6.022	0.28	5.729	6.040	0.31
16-PAM	Gray	(2,1,0,3)	3	11.370	8.377	8.881	0.50	8.360	8.906	0.55
16-PAM	SP	(2,1,0,3)	3	11.361	8.368	8.881	0.51	8.351	8.906	0.56
32-PAM	Gray	(1,3,2,0,4)	4	15.060	11.098	11.835	0.74	11.081	11.869	0.79
32-PAM	SP	(2,3,0,1,4)	4	15.061	11.099	11.835	0.74	11.082	11.869	0.79
R=1/4										
4-PAM	Gray	(1,0)	1	−0.801	2.227	2.599	0.37	2.209	2.599	0.39
4-PAM	SP	(0,1)	1	−0.804	2.224	2.599	0.38	2.206	2.599	0.39
8-PAM	Gray	(2,0,1)	2	2.344	3.611	4.346	0.74	3.593	4.349	0.76
8-PAM	SP	(1,0,2)	2	2.342	3.609	4.346	0.74	3.591	4.349	0.76
16-PAM	Gray	(2,1,0,3)	3	5.082	5.099	6.028	0.93	5.082	6.036	0.95
16-PAM	SP	(3,1,0,2)	3	5.083	5.101	6.028	0.93	5.083	6.036	0.95
32-PAM	Gray	(1,3,2,0,4)	4	7.633	6.681	7.700	1.02	6.664	7.713	1.05
32-PAM	SP	(3,2,0,1,4)	4	7.625	6.673	7.700	1.03	6.656	7.713	1.06

**Table 5:** Asymptotic DE thresholds of 8-PAM under optimal and worst-case pilot designs ($E_{s}/N_{0}$ in dB).

		R=1/2			R=1/4		
Labeling	d	Optimal	Worst-Case	Loss	Optimal	Worst-Case	Loss
Gray	(0,2,1)	7.522	8.993	1.47	2.360	3.823	1.46
Gray	(1,0,2)	7.520	10.037	2.52	2.351	6.007	3.66
SP	(1,0,2)	7.490	8.236	0.75	2.342	3.074	0.73
SP	(0,1,2)	7.496	7.550	0.05	2.361	2.782	0.42

**Table 6:** DE thresholds of the irregular LDPC codes at $R_{\mathrm{eff}}=0.498$ (T=249 for 4-PAM, T=498 for 8-PAM), evaluated with 1000 iterations and a target error probability of $10^{-8}$. In the shortened BICM baseline, the shortened bits are treated as perfectly reliable variable nodes and are distributed over the degree classes in proportion to their node fractions.

	System	$E_{s}/N_{0}$ [dB]	$E_{b}/N_{0}$ [dB]	Gain [dB]
4-PAM	BICM, shortened	2.555	2.573	—
	DBICM, d=(1,0)	2.458	2.475	0.098
8-PAM	BICM, shortened	7.012	5.269	—
	DBICM, d=(1,0,2)	6.493	4.749	0.520

**Table 7:** Node-perspective degree distributions $\Lambda^{\left(i\right)}$ and class-average degrees ${\bar{d}}_{v}^{\left(i\right)}$ of the irregular codes. Every class comprises $n_{c}/m$ variable nodes, and the overall average degree is ${\bar{d}}_{v}=3.5$ in all cases.

Class	$\Lambda_{2}$	$\Lambda_{3}$	$\Lambda_{4}$	$\Lambda_{8}$	$\Lambda_{10}$	${\bar{d}}_{v}^{\left(i\right)}$
4-PAM, *one profile per bit level, used unchanged by both systems*						
i=0	0.2892	0.4698	0.2245	0	0.0164	3.05
i=1	0.5988	0.1799	0	0	0.2213	3.95
overall	0.4440	0.3249	0.1123	0	0.1188	3.50
8-PAM, *three constituent profiles*						
*A*	0.5000	0.5000	0	0	0	2.50
*B*	0	0.5000	0.3750	0.1250	0	4.00
*C*	0.7500	0	0	0	0.2500	4.00
overall	0.4167	0.3333	0.1250	0.0417	0.0833	3.50
8-PAM assignment		i=0	i=1	i=2		
BICM (delays (0,0,0))		*A*	*B*	*C*		
DBICM, d=(1,0,2)		*B*	*A*	*C*		

**Figure A1:**
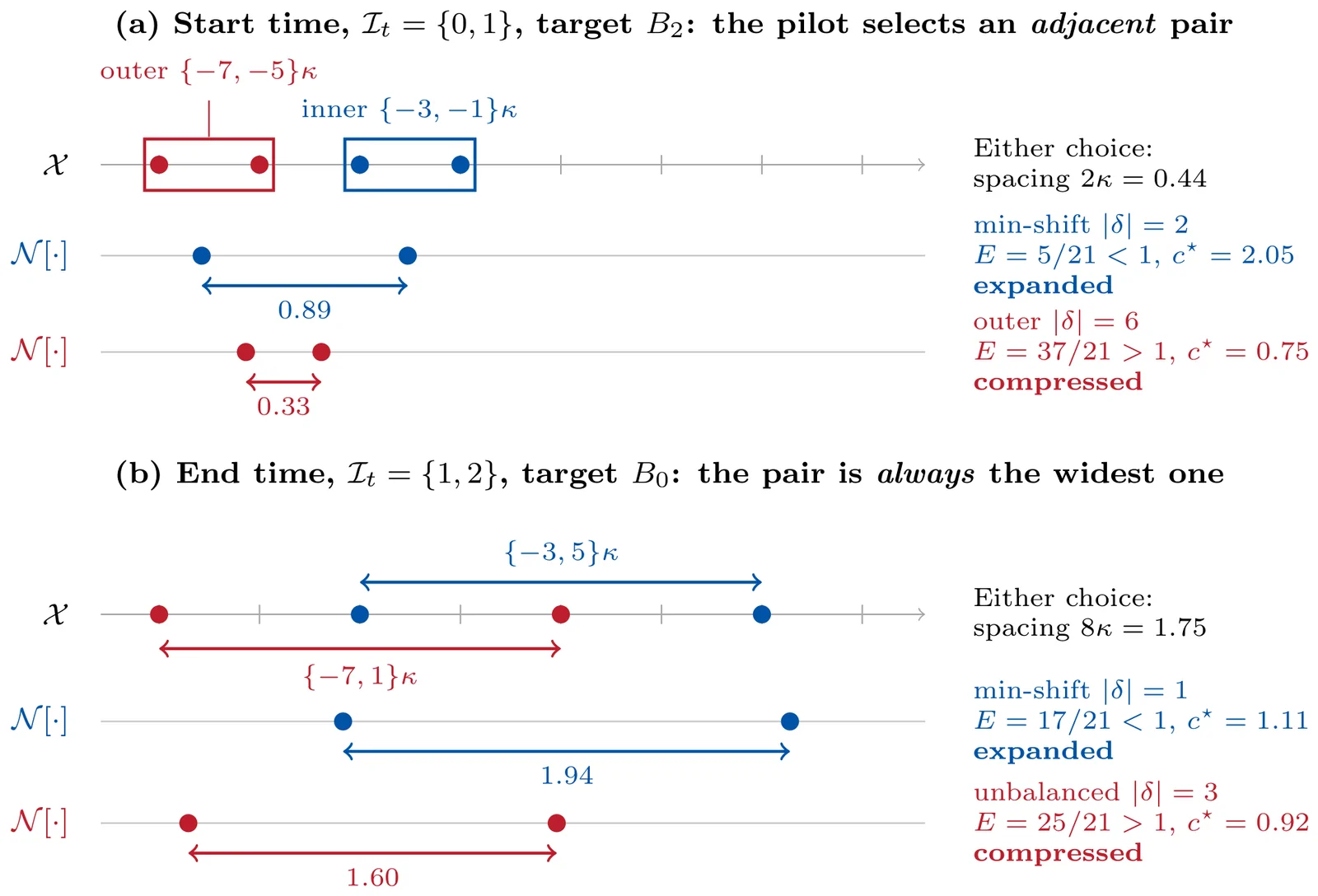
Sub-constellations retained by the pilots under SP labeling for 8-PAM with d=(2,1,0), before (X) and after ($N_{\mathrm{sub}}\left[\cdot \right]$) unit-power normalization. The constellation geometry and bit mapping follow Figure 1b, with $\kappa =1/\sqrt{21}$. (**a**) Start boundary ($I_{t}=\left\{0,1\right\}$, target bit $B_{2}$): every pilot assignment leaves an adjacent pair with spacing 2κ, where the minimum-shift pilot minimizes the distance to the origin. (**b**) End boundary ($I_{t}=\left\{1,2\right\}$, target bit $B_{0}$): every pilot assignment leaves a pair with spacing 8κ, where the minimum-shift pilot balances the points symmetrically around the origin. In both regions, the minimum-shift pilot achieves $c^{⋆}\geq 1$, and the normalized point spacing matches the quantity analyzed in Lemma 3.

## Data Availability

The original contributions presented in this study are included in the article. Further inquiries can be directed to the corresponding author.

## Appendix A. Pilot Assignments Used in This Paper

This appendix tabulates the boundary pilot assignments underlying the capacity, density evolution, and simulation results reported in this paper. Because the pilot index sets $I_{t}$ are fully determined by the delay vector via (10), only the pilot *values* require specification, in analogy with reporting a convolutional encoder by its generator words. Each assignment is therefore encoded as two binary words: the start-side word $w_{s}=p_{0}\;|\;p_{1}\;\left|\;\cdots \;\right|\;p_{d_{\max}-1}$ and the end-side word $w_{e}=p_{T}\;|\;p_{T+1}\;\left|\;\cdots \;\right|\;p_{T_{s}-1}$, where each group $p_{t}={\left(p_{t,i}\right)}_{i\in I_{t}}$ lists the pilot values at symbol time *t* in ascending order of the bit index *i*. For a staircase delay vector, each side comprises $\sum_{i}d_{i}=m\left(m-1\right)/2$ bits. For example, for 8-PAM with d=(1,0,2), the word $w_{s}=00|0$ encodes $p_{0,0}=0$, $p_{0,2}=0$, and $p_{1,2}=0$.

Table A1 lists the optimized assignments under Gray labeling obtained by the exhaustive search of Section 6.3, covering the delay vectors of Table 2 and Table 4. Under SP labeling, no search is required, because the optimized pilots are the minimum-shift assignments of Definition 4. Table A2 lists one minimizer per delay vector for concreteness, and by Remark 3, every other minimizer yields identical boundary rates and margins. Table A3 lists the worst-case assignments used in Table 5 and Figure 7. Under SP labeling, the worst-case assignment is the *maximum-shift* one, which is the mirror image of Definition 4 and is attained by setting all pilot bits within each boundary symbol time to a common value. By Lemmas 2 and 3, this assignment uniformly minimizes every boundary margin, and the all-zero assignment shown in Table A3 is one such representative. Together with the closed-form constellation and labeling specification in Section 2.2, these tables fully specify the transmitted signals in all boundary regions.

**Table A1:** Optimized boundary pilot assignments under Gray labeling for the delay vectors of Table 2 and Table 4 (all six staircase vectors are included for 8-PAM). Each entry lists the start-side word $w_{s}=p_{0}\;\left|\;\cdots \;\right|\;p_{d_{\max}-1}$ and the end-side word $w_{e}=p_{T}\;\left|\;\cdots \;\right|\;p_{T_{s}-1}$, where each group $p_{t}={\left(p_{t,i}\right)}_{i\in I_{t}}$ is ordered by ascending bit index *i* and the pilot index sets $I_{t}$ are determined by the delay vector via (10).

Constellation	d	$w_{s}$ (Start)	$w_{e}$ (End)
4-PAM	(0,1)	0	0
	(1,0)	0	0
8-PAM	(0,1,2)	00|1	0|00
	(0,2,1)	00|0	0|00
	(1,0,2)	00|0	0|00
	(1,2,0)	00|0	0|00
	(2,0,1)	00|0	0|00
	(2,1,0)	00|0	1|00
16-PAM	(0,2,3,1)	000|00|1	0|10|000
	(2,1,0,3)	000|10|0	1|00|000
	(3,1,0,2)	000|10|0	1|00|000
32-PAM	(0,2,3,4,1)	0000|000|11|1	0|00|000|0000
	(0,3,2,4,1)	0000|010|01|1	0|00|000|0000
	(1,2,3,4,0)	0000|010|11|1	0|00|000|0000
	(1,3,2,0,4)	0000|000|00|0	1|01|011|0000
	(2,3,1,0,4)	0000|000|00|0	1|11|011|0000
	(3,2,1,0,4)	0000|000|00|0	1|11|000|0000
	(4,1,2,0,3)	0000|000|00|0	1|01|000|0000
	(4,2,1,0,3)	0000|000|00|0	1|11|000|0000
	(4,3,1,0,2)	0000|000|00|0	1|11|110|0000

**Table A2:** Minimum-shift pilot representatives (Definition 4) under SP labeling for the delay vectors used in this paper, in the same notation as Table A1. By Remark 3, every other minimizer yields identical boundary rates and margins.

Constellation	d	$w_{s}$ (Start)	$w_{e}$ (End)
4-PAM	(0,1)	0	0
8-PAM	(0,1,2)	01|0	0|01
	(0,2,1)	01|0	0|01
	(1,0,2)	01|0	0|01
	(1,2,0)	01|0	0|01
	(2,0,1)	01|0	0|01
	(2,1,0)	01|0	0|01
16-PAM	(2,1,0,3)	011|01|0	0|01|011
	(3,1,0,2)	011|01|0	0|01|011
32-PAM	(2,3,0,1,4)	0111|011|01|0	0|01|011|0111
	(3,2,0,1,4)	0111|011|01|0	0|01|011|0111

**Table A3:** Worst-case pilot assignments for 8-PAM used in Table 5 and Figure 7, in the same notation as Table A1. Under SP labeling, the worst-case assignment is the maximum-shift one, for which all pilot bits equal 0 (equivalently, all equal 1).

Labeling	d	$w_{s}$ (Start)	$w_{e}$ (End)
Gray	(0,2,1)	00|1	0|01
Gray	(1,0,2)	01|0	1|01
SP	(0,1,2)	00|0	0|00
SP	(1,0,2)	00|0	0|00

## Appendix B. Proof of Theorem 1 and Example

**Proof of Theorem** **1.** Let $t=\psi_{i}\left(k\right)$ denote the boundary symbol time associated with the boundary pair $\left(k,i\right)\in D_{\mathrm{bd}}$. By (24), the rate margin is expressed as

(A1) $$\Delta_{k,i}=\underset{\mathrm{boundary}:\;\mathrm{fixed}\;\mathrm{via}\;N_{\mathrm{sub}}}{\underset{︸}{I_{X_{P_{t}\left(a^{⋆}\right)}}\;\left(B_{t,i};Y_{t}\;|\;H_{t,i}^{\mathrm{bd}}\right)}}-\underset{\mathrm{middle}:\;\mathrm{conditioning}\;\mathrm{only}}{\underset{︸}{C_{i}^{\mathrm{mid}}\left(d\right)}}.$$

*Middle-region term (native scale, c=1).* The conditioning bits in the middle-region achievable rate $C_{i}^{\mathrm{mid}}\left(d\right)$ in (14) are independent uniform random variables. The term is of the conditioning-only form (C) of Section 5.3, so that the input alphabet is the full constellation X. Furthermore, by Lemma 2, averaging over all translated sub-constellations confirms that the target-bit achievable rate is identical to that of the free component *S* observed at the native power scale (c=1). Hence, the middle-region term evaluates exactly to $I^{\left(1\right)}\left(B_{t,i};Y_{t}|H_{t,i}\right)$. *Boundary-region term (re-normalized scale, $c=c^{⋆}$).* The pilot constraint $P_{t}\left(a^{⋆}\right)$ restricts the channel input alphabet to $N_{\mathrm{sub}}\;\left[X_{t}\left(a^{⋆}\right)\right]=c^{⋆}\left(a^{⋆}\right)\;\kappa \;S_{\mathrm{bd}}$ (up to an exact Euclidean translation), where $S_{\mathrm{bd}}$ denotes the free summation over all bit levels that are neither fixed by pilots nor conditioned upon at symbol time *t*. The composition of $S_{\mathrm{bd}}$ differs between the two boundary regions according to (21), because by Lemma 1, the active pilot positions lie on a definite side of the target level for every boundary pair (k,i), regardless of *k*. At a *start-boundary* time, the union of fixed and conditioned levels is $I_{t}\cup H_{t,i}^{\mathrm{bd}}=\left\{\;j:\;d_{j}>d_{i}\;\right\}$, which coincides with the index set of the middle-region side-information bits $H_{t,i}$. Consequently, $S_{\mathrm{bd}}=S$, and the boundary term evaluates to $I^{\left(c^{⋆}\right)}\left(B_{t,i};Y_{t}|H_{t,i}^{\mathrm{bd}}\right)$ over the *identical* free component *S*. At an *end-boundary* time, the corresponding union is $\left\{\;j:\;d_{j}>d_{i}\;\right\}\cup I_{t}$, so that $S_{\mathrm{bd}}$ is obtained from *S* by additionally fixing the low-delay pilot levels $I_{t}\subseteq \left\{\;j:\;d_{j}<d_{i}\;\right\}$. *Scale bound $c^{⋆}\geq 1$.* Because the scale bound depends solely on the index set $I=I_{t}$ and the minimization in (33), it holds universally across both boundary regions. The energy normalization identity $1=\kappa^{2}\sum_{i=0}^{m-1}4^{\;m-1-i}$ and (31) yield

$$1-E_{s}^{\mathrm{rel}}=\kappa^{2}\;\;\sum_{i\in I}\;4^{\;m-1-i}\;\geq \;\kappa^{2}\;4^{\;m-1-i_{\min}},$$

where $i_{\min}≜\min_{i\in I}i$ indexes the most significant fixed bit (which maximizes $2^{\;m-1-i}$). The minimum-shift condition in (33) ensures that $\delta {\left(a^{⋆}\right)}^{2}\leq 4^{\;m-1-i_{\min}}\leq \sum_{i\in I}4^{\;m-1-i}$. Substituting this inequality into (32) yields $\kappa^{2}\delta {\left(a^{⋆}\right)}^{2}+E_{s}^{\mathrm{rel}}\leq 1$, which proves that $c^{⋆}\geq 1$. *Rate margin for start-boundary pairs.* Both the boundary and middle terms represent the identical functional of the same underlying free component *S*, evaluated at effective scaling factors $c=c^{⋆}$ and c=1, respectively. Since $c^{⋆}\geq 1$, Lemma 3 implies that the boundary achievable rate is no smaller than the middle-region rate, establishing $\Delta_{k,i}\geq 0$ in (34). *Rate margin for end-boundary pairs.* For end-boundary pairs, the two terms are governed by different free subsets because $S_{\mathrm{bd}}⊊S$. The margin comparison is established by chaining two inequalities. First, Lemma 3 with $c^{⋆}\geq 1$, applied to the restricted component $S_{\mathrm{bd}}$, gives

$$I^{\left(c^{⋆}\right)}\left(B_{t,i};Y_{t}\;|\;H_{t,i}^{\mathrm{bd}}\right)\;\geq \;I^{\left(1\right)}\left(B_{t,i};Y_{t}\;|\;H_{t,i}^{\mathrm{bd}}\right).$$

Second, $S_{\mathrm{bd}}$ differs from *S* solely in that the bit levels at indices $I_{t}$ are fixed constants rather than uniform random variables. By Lemma 2, fixing a pilot value acts as conditioning up to a Euclidean translation, and conditioning on independent bits cannot reduce the mutual information, so that

$$I^{\left(1\right)}\left(B_{t,i};Y_{t}\;|\;H_{t,i}^{\mathrm{bd}},{\left\{B_{t,j}\right\}}_{j\in I_{t}}\right)\;\geq \;I^{\left(1\right)}\left(B_{t,i};Y_{t}\;|\;H_{t,i}\right),$$

where the left-hand side is the rate evaluated over $S_{\mathrm{bd}}$ and the right-hand side is the rate evaluated over *S*, and where $H_{t,i}^{\mathrm{bd}}=H_{t,i}$ at end-boundary times. Chaining these two inequalities confirms that $\Delta_{k,i}\geq 0$. *Equality and strict positivity conditions.* For a start-boundary pair with $|I_{t}|=1$, $\delta \left(a\right)=\pm 2^{\;m-1-i^{\prime }}$, where $i^{\prime }$ is the unique fixed level, which yields $\kappa^{2}\delta^{2}=1-E_{s}^{\mathrm{rel}}$, so that $c^{⋆}=1$ and equality holds identically. For start-boundary pairs with $|I_{t}|\geq 2$, the strict inequality $\delta {\left(a^{⋆}\right)}^{2}<\sum_{i\in I_{t}}4^{\;m-1-i}$ holds automatically. Since the weights $2^{\;m-1-i}$ for $i\in I_{t}$ are distinct powers of two, the sign pattern that assigns the most significant fixed bit opposite to all remaining bits achieves $\left|\delta \right|\leq 2^{\;m-1-i_{\min}}-1$, and the minimizer therefore satisfies $\delta {\left(a^{⋆}\right)}^{2}<4^{\;m-1-i_{\min}}\leq \sum_{i\in I_{t}}4^{\;m-1-i}$. Therefore, $c^{⋆}>1$, rendering the margin strictly positive prior to capacity saturation via Lemma 3. For end-boundary pairs, the second inequality in the chain is strict whenever the fixed low-delay levels contribute a nonzero separation to *S*, which is always the case for SP labeling, so that the margin is strictly positive prior to saturation. □

### Example (*8*-PAM, ***d** = (2, 1, 0)*)

Consider the reversed staircase delay vector d=(2,1,0) and the level-2 (LSB) bit of codeword k=0, which is transmitted at $t=\psi_{2}\left(0\right)=0$. At this symbol time, $I_{0}=\left\{0,1\right\}$, so that the two most significant bit positions are fixed by pilots, and $\kappa^{2}=1/21$. The constellation and its labeling correspond to those in Figure 1b, whose normalized amplitudes are ±0.22,±0.65,±1.09, and ±1.53. With $\delta \left(a\right)=2^{2}\left(2a_{0}-1\right)+2^{1}\left(2a_{1}-1\right)$ from (30) for m=3, the minimum-shift assignment $a^{⋆}\in \left\{\left(0,1\right),\left(1,0\right)\right\}$ yields |δ|=2. It selects the *inner* sub-constellation {−3,−1} or {1,3}, whose average energy is $E(X_{t}\left(a^{⋆}\right))=5/21$ and whose scaling factor is $c^{⋆}=\sqrt{21/5}\approx 2.05>1$. An outer assignment with |δ|=6, which selects {−7,−5} or {5,7}, yields $c^{⋆}=\sqrt{21/37}<1$ by contrast. Table A4 lists the resulting rate margins $\Delta_{k,i}$ under the DBICM model. The minimum-shift pilot maintains Δ≥0 at all SNRs, whereas the outer pilot yields Δ<0, which illustrates that the pilot selection is decisive and that Theorem 1 provides the favorable assignment in closed form.

**Table A4:** Rate margin $\Delta_{k,i}$ (in bits/symbols) for 8-PAM with SP labeling (d=(2,1,0)) in the start region for the target LSB (i=2), where the pilots fix the two most significant bits (MSBs) at t=0. The column “Middle” lists the per-level middle-region term $C_{2}^{\mathrm{mid}}\left(d\right)$ of (14) for the target bit, and $\Delta =\mathrm{boundary}-C_{2}^{\mathrm{mid}}$.

$E_{s}/N_{0}$ (dB)	Middle	Δ (Min-Shift $a^{⋆}$)	Δ (Outer Pilot)
−5	0.0214	+0.0645	−0.0092
0	0.0656	+0.1761	−0.0276
5	0.1895	+0.3749	−0.0757
10	0.4702	+0.4426	−0.1610
15	0.8464	+0.1528	−0.1782
20	0.9958	+0.0042	−0.0354

Figure A1 illustrates the geometric intuition behind these numerical values alongside their end-region counterparts, highlighting the structural asymmetry between the two boundaries. At a start symbol time, shown in panel (a), the unconstrained bit is the LSB, so that the surviving signal pair is always an *adjacent* pair with Euclidean spacing 2κ, and the pilot assignment determines only *where* on the constellation axis this pair is located. The minimum-shift pilot retains the innermost pair, whose sub-constellation energy 5/21<1 causes power re-normalization to expand the spacing from 0.44 to 0.89, whereas the outer pair is compressed to 0.33. At an end symbol time, shown in panel (b), where $I_{t}=\left\{1,2\right\}$ and the target bit is $B_{0}$, the unconstrained bit is the MSB, so that the surviving pair is always the *widest* pair with spacing 8κ regardless of the pilot values, and only its offset from the origin varies. The minimum-shift setting with |δ|=1 results in a balanced pair that is expanded ($c^{⋆}=1.11$), whereas the unbalanced setting with |δ|=3 is compressed ($c^{⋆}=0.92$). The end boundary is inherently more resilient than the start boundary because, in addition to the power scaling, it benefits from the removal of interference established in the proof of Theorem 1. The start boundary possesses no such mechanism, which explains why an outer pilot yields strictly negative margins across all SNRs in that region.
